# Efficacy and tolerability of D-β-hydroxybutyrate ester in patients with amyotrophic lateral sclerosis (KETO-ALS)–a randomised, placebo-controlled, double-blind study

**DOI:** 10.1016/j.eclinm.2026.104272

**Published:** 2026-10-26

**Authors:** Christine Herrmann, Luisa Jagodzinski, Zeynep Elmas, Tabea Herschmann, Elke Fröhlich, Franziska Bachhuber, Tanja Ruschitzka, Frank Willkomm, Özlem Parlak, Ulrike Weiland, Felicitas Becker, Lars Richter, Maximilian Wiesenfarth, Zeljko Uzelac, Sebastian Michels, Simon Witzel, Hayrettin Tumani, Jochen H. Weishaupt, Ludwig Maier, Benjamin Mayer, Jens Dreyhaupt, Joachim Schuster, Johannes Dorst

**Affiliations:** a Department of Neurology, University of Ulm, Ulm, Germany; b German Center for Neurodegenerative Diseases (DZNE), Ulm, Germany; c Institute of Epidemiology and Medical Biometry, University of Ulm, Ulm, Germany; d Ulm University Hospital Pharmacy, University of Ulm, Ulm, Germany

**Keywords:** Amyotrophic lateral sclerosis, Beta hydroxybutyrate ester, Oral ketone bodies, Neurofilament light chain

## Abstract

**Background:**

Hypermetabolism and weight loss are prominent features of amyotrophic lateral sclerosis (ALS). Here, we sought to target the energy deficit in ALS by applying oral ketone bodies as high-energetic substrates.

**Methods:**

Patients with probable or definite ALS were enrolled to this randomised, placebo-controlled, parallel-group, double-blind, monocentre phase 2 trial. Eligible patients had a decline of ALS functional rating scale revised (ALSFRS-R) of at least 0.33 points/month. They were randomly assigned (1:1) to receive D-β-hydroxybutyrate ester (βHB, 30 g/day) or placebo. Primary endpoint was change of neurofilament light chain (NfL) per month, measured as slope over 6 months. Multiple linear regressions models were applied to adjust for NfL, age, BMI, and slow vital capacity (SVC) at baseline. The trial is registered with clinicaltrials.gov, number NCT04820478.

**Findings:**

Between April 5, 2022, and February 12, 2025, 81 patients were randomly assigned to receive βHB (n = 40) or placebo (n = 41) and included in the intention-to-treat analysis. In the unadjusted analysis, the primary endpoint NfL slope was negative, showing a decline of −0.5 pg/mL/month (IQR −3.2 to 2.0, minimum −11.2, maximum 10.4) in the βHB group and a stable course of 0.0 pg/mL/month (IQR −1.7 to 3.3, minimum −6.2, maximum 49.0) in the placebo group (p = 0.094). In the adjusted analysis, the NfL slope showed a benefit for the βHB group (−1.5 pg/ml/month in βHB vs. +2.8 pg/ml/month in placebo, mean difference −4.2 pg/ml/month, 95% CI - ∞ to −1.1, p = 0.014). When using a mixed effect model, this effect was not statistically different (−0.4 pg/ml/month in βHB vs. +0.3 pg/ml/month in placebo, mean difference −0.7 pg/ml, month, p = 0.30). In the unadjusted analysis, ALSFRS-R showed a faster decline in the verum group (−1.4 points/month (IQR −2.1 to −0.6) vs. −0.8 points/month (IQR −1.2 to −0.2), p = 0.020). No significant difference between βHB and placebo was found regarding clinical endpoints such as ALSFRS-R or BMI in the adjusted analysis. βHB was well tolerated, with similar frequency of adverse events across both groups.

**Interpretation:**

Whether the effects on NfL can be confirmed and translated into meaningful clinical effects, remains to be clarified by a larger phase 3 trial with extended treatment duration. βHB was well tolerated.

**Funding:**

This was an investigator-initiated study, funded by a private donation.

**Research in context**

**Evidence before this study**

We searched PubMed and clinicaltrials.gov for reviews, observational studies, clinical trials, and cohort studies investigating efficacy and/or safety of ketone bodies or ketogenic diet in ALS published up to October 12th, 2025, using the terms “amyotrophic lateral sclerosis” or “ALS” in combination with “ketone”, “ketogenic”, and “beta hydroxybutyrate”. After reviewing the abstracts, we identified 5 reviews dealing with ketone bodies or ketogenic diet in ALS, 1 case report of ketogenic diet in ALS, 2 cohort studies investigating serological parameters related to ketogenic diet, and 2 animal studies applying ketogenic diet in mice. One open label trial with ketogenic diet in ALS (NCT01016522) has been terminated after inclusion of one patient. We found no randomised, placebo-controlled trials with ketogenic interventions.

**Added value of this study**

This randomised, placebo-controlled trial investigating efficacy and safety of oral ketone bodies provided evidence that D β-hydroxybutyrate ester is well tolerated while also providing preliminary efficacy data.

**Implications of all the available evidence**

Whether the effect on NfL can be translated to meaningful clinical effects, must be confirmed by a trial with larger sample size investigating clinical outcome parameters over a longer period.

## Introduction

Weight loss is a common phenomenon and a prominent negative prognostic factor in amyotrophic lateral sclerosis (ALS).^1^, ^2^ Body mass index (BMI) decline already begins up to 10 years prior to disease onset^3^ and is associated with worse prognosis.^4^ Although the pathophysiological mechanism of weight loss in ALS is multicausal and not fully understood, an increased resting energy expenditure (REE), commonly referred to as hypermetabolism, seems to play an important role. Studies using indirect calorimetry revealed that a significant share of patients with sporadic ALS exhibit an increased REE,^5^, ^6^, ^7^, ^8^, ^9^ which seems to persist during the whole course of disease with a slight decrease of REE in later disease stages.^5^, ^6^ The hypermetabolic state is already evident in the presymptomatic ALS mouse model,^10^ and indirect evidence based on cross-sectional BMI, calorimetry, and nutritional data suggests that increased REE might also contribute to premorbid weight loss in ALS patients.^11^, ^12^ Since body weight as an important prognostic factor is directly influenced by calorie intake and expenditure, it is commonly assumed that hypermetabolism also affects prognosis, although a causal relationship has not been established. Moreover, including increased lipid mobilisation and fatty-acid oxidation, as well as a deficient utilization of carbohydrates, has been described in ALS.^13^, ^14^

Mitochondrial dysfunction, particularly impairment of complex I of the respiratory chain, is one of two major hypotheses (alongside a hypothalamic atrophy/dysfunction) that play a major role in the origin of hypermetabolism, resulting in an insufficient production of adenosine triphosphate (ATP).^15^, ^16^, ^17^, ^18^ In a randomised, controlled study (LIPCAL-ALS), the additional intake of 405 kcal/day and 45 g fat per day showed a reduction of neurofilament light chain (NfL) serum levels and longer survival in fast-progressing patients over 18 months compared to placebo.^19^, ^20^

Based on the pathologic metabolic alterations found in ALS, a ketogenic diet, containing a low-carbohydrate, high-fat nutrition, represents another logical candidate for a therapeutic intervention, as ketone bodies constitute high-energetic substrates, which can cross the blood brain barrier via MCT1, a specific physiological transporter. Under “western” nutrition, which is mainly based on carbohydrates, the central nervous system utilises glucose as the main substrate to produce ATP. However, under conditions associated with glucose shortage such as fasting, a ketogenic diet, or even during sleep or excessive exercising,^21^ the liver synthesises ketone bodies (β-hydroxybutyrate, acetoacetate, and acetone) from fatty acids, which provide a significantly higher amount of ATP per mole compared to glucose.^22^ Interestingly, astrocytes, but not neurons, are also able to synthesise ketone bodies.^23^, ^24^ Recent studies exploring metabolic alterations in ALS patients suggested a mobilisation of fatty acids from the adipose tissue and production of high-energetic ketone bodies as a compensatory mechanism to counter the energy deficit already in early stages of the disease.^25^

In the SOD1-G93A mouse model of ALS, animals treated with a ketogenic diet gained body weight and showed a significantly slower decline of motor function, while maintaining a higher number of motor neurons in the ventral horn of the spinal cord compared to controls.^26^ When isolating mitochondria from the same mice and adding D-β-3-hydroxybutyrate, ATP synthesis was increased.^26^ Another study in the same mouse model also demonstrated a longer maintenance of motor function and improved survival in mice that were fed with a ketogenic diet and additional amino acids compared to a standard diet with the same additional amino acids.^27^ Ketogenic diet improved mitochondrial function in a mouse model via activation of PGC1α, a transcriptional co-activator for regulation of mitochondrial biogenesis.^28^ Moreover, D-β-hydroxybutyrate restored function of complex I and improved ATP production in cultured neurons, in which this complex was pharmaceutically blocked.^29^ Since glucose metabolism has been shown to be impaired in ALS,^14^ and fatty acids cannot be metabolised by motor neurons due to their inability to cross the blood–brain-barrier, ketone bodies might constitute the optimal alternative energy substrate in this specific situation.

However, a ketogenic diet is difficult to establish in ALS patients as it requires a long-term change of eating habits, which is hindered by dysphagia, a rapidly declining general condition and reduced life expectancy. Moreover, restrictive choice of food associated with a ketogenic diet may carry the risk of reducing energy intake and aggravating weight loss. Therefore, the direct application of ketone bodies may constitute an effective and practicable alternative compared to a ketogenic diet. In recent years, it has been shown, that orally applied ketone bodies, either via salts or esters, significantly increase ketone body serum levels, which sustain for several hours.^30^, ^31^ Moreover, it has been repeatedly demonstrated that the intake of oral ketone bodies, even in higher dosages, is safe and well tolerated.^31^, ^32^, ^33^ In a case series with two ALS patients, ketone esters were well tolerated and significantly increased capillary βHB levels for one to 2 h.^34^

For this study, we hypothesised that the energy deficit in ALS can be ameliorated by administering ketone bodies as a high-energetic substrate, and that this intervention is able to slow down disease progression as indicated by decline of NfL serum levels. The overall concept of the study was to focus on NfL first, and to perform a follow-up study with longer treatment duration to evaluate functional endpoints, in case positive signals were received. By maintaining sufficient caloric intake and, at the same time, providing an additional, more efficient substrate for generating energy, we hypothesised that this alternate fuel can be beneficially utilised in damaged neurons by enabling reparation and anabolic processes to counteract cachexia and weight loss. Therefore, in this randomised, placebo-controlled trial, we explored the effect of an orally administered D-β-hydroxybutyrate ester (βHB) on NfL serum levels over an intervention period of six months.^35^, ^36^

## Methods

### Study design

This study was a prospective, randomised, double-blind, placebo-controlled study of oral βHB-ester as add-on therapy to standard treatment, including riluzole and high-caloric nutrition, over an intervention period of six months. It was conducted at the Department of Neurology of Ulm University, Ulm, Germany, and was done in accordance with the Declaration of Helsinki, International Conference on Harmonisation Guideline for Good Clinical Practice. The study protocol was approved by the independent ethics committee of Ulm University, Germany (approval number 145/21). All study participants provided written informed consent. For review of safety results, an independent rater performed an interim safety analysis, when 50% of patients had completed the study. During this interim safety analysis, no concerns regarding continuation of the study were raised. The trial was registered with ClinicalTrials.gov, number NCT04820478 prior to the first enrolment.

The study was performed over six months including three on-site visits (combined screening/baseline visit, after three, and after six months) and two phone visits to check tolerability and compliance (after three days and two weeks).

### Patients

Patients with possible, probable (clinically or laboratory-supported) or definite ALS according to the revised version of the El Escorial World Federation of Neurology criteria^37^ were considered for enrolment into the study. Patients featured a loss of ALS functional rating scale revised (ALSFRS-R^38^) of ≥0.33 points per month since onset (defined as presence of first paresis) based on the formula: (48 – score at screening visit)/(Months between onset and screening visit). This criterion assured that patients with very slow progression did not enter the study, since disease progression of such patients was considered too slow to display a significant change of NfL within the timeframe of the study. Patients received a stable dosage of riluzole for at least four weeks prior to inclusion. Patients with severe gastro-oesophageal reflux disorder or known metabolic disorders, which constitute contraindications against ketogenic diet (hyperinsulinism, pyruvate decarboxylase deficit, disturbance of fatty acid oxidation, disturbance of gluconeogenesis, and acute porphyria) were excluded.

### Randomisation and masking

At screening, eligible patients were enrolled into the study and randomly assigned (1:1) to the intervention or the placebo group by receiving the next consecutive randomisation number according to their stratum. Stratification was done according to slow (≤0.77 points of loss of ALSFRS-R/month) or fast (>0.77 points of loss of ALSFRS-R/month) disease progression and the presence or absence of weight loss within six months prior to baseline. The decision to estimate disease progression based on ALSFRS-R pre-baseline instead of NfL was made because stratification based on NfL would have prevented the opportunity to perform screening and baseline in a combined visit. A binary criterion for weight loss was implemented as the presence of weight loss has repeatedly been reported as an independent negative prognostic factor.^1^ An independent person at the Institute of Epidemiology and Medical Biometry, University of Ulm, Germany, generated the randomisation list, using a validated system, which involves a pseudorandom number generator to ensure that the resulting treatment sequence will be both reproducible and nonpredictable. The randomisation was performed by the Ulm University Hospital pharmacy.

Patients and site personnel were masked to treatment allocation. Serum β-hydroxybutyrate, acetoacetate, total ketones, and urinary acetone were measured, but respective results were not communicated to the study team. Instead, all results were collected and stored by an independent person until the end of the study to maintain blinding.

The oral βHB (KE4®, KetoneAid, Falls Church, USA) is commercially available. Placebo solution was manufactured by the Ulm University Hospital pharmacy. The composition of both solutions is listed in Supplementary Table S1. Blinding, packaging, and labelling were performed by the Ulm University Hospital pharmacy according to the randomisation list.

### Procedures

Patients in the intervention group received 20 ml (10 g) of βHB 3 times daily. Patients were instructed to take the study medication in the morning, in the afternoon, and in the evening with maximum distance (at least 1.5 h) to meals, since food intake (especially carbohydrates) may lower ketone body serum levels.^31^ The applied dosage was based on tolerability and ketone body blood levels as determined during a preceding test with various dosages in six healthy probands and two patients, described in detail in the study protocol and a previous case series.^34^ Patients in the placebo group were administered 20 ml of matching placebo 3 times daily. Verum and placebo had similar amounts of calories (48 kcal vs. 32 kcal per dosage). Data about appetite and daily food intake were collected by applying a standardised nutrition and appetite score (Council of Nutrition Appetit Questionnaire (CNAQ)^39^) and a nutrition questionnaire (Ulm Nutrition Questionnaire^19^), evaluating eating habits and composition of meals. By applying these measures, we aimed to identify a potential bias caused by a change of eating habits or appetite due to the intervention. Patients were allowed to receive high-caloric nutritional supplements as part of standard care during the study.

### Outcomes

Primary endpoint was change of NfL serum levels after 6 months compared to baseline, measured as slope (change per month). Secondary outcomes were change of resting energy expenditure (REE, expressed as metabolic ratio (MR), which constitutes the quotient of the measured resting metabolic rate and the calculated resting metabolic rate based on age, sex, height, and weight) according to the Harris-Benedict equation^40^ measured by indirect calorimetry and change of body composition (fat mass, body cell mass, extracellular mass, and phase angle) measured by bioelectrical impedance analysis (BIA). Moreover, survival (time to death or tracheostomy) after 6 months as well as changes of ALSFRS-R, BMI, slow vital capacity (SVC), individual quality of life (EQ-5D-5L^41^), ketone bodies in serum and urine, and parameters related to lipid metabolism, all measured as slope (change per month), were analysed. Since there are no ALS-specific scales, appetite was evaluated with the CNAQ as the best validated appetite score in clinical practice. Additionally, absolute differences in βHB in serum and acetone in urine (βHB is metabolised and eliminated via acetone) were analysed to assess compliance and the association between these parameters and NfL. Safety analysis included frequency of adverse events and serious adverse events as well as laboratory safety parameters (clinical chemistry and haematology).

### Statistical analysis

The sample size calculation was based on a subgroup analysis of the precursor LIPCAL-ALS study^20^ as represented by the proposed inclusion/exclusion criteria (ALSFRS-R slope pre-baseline ≥0.33 points), because LIPCAL-ALS represented the thematically closest existing study (nutritional intervention targeting hypermetabolism and weight loss). Assuming the same effect size on NfL as seen in this subgroup in LIPCAL-ALS (mean difference in the NfL slopes of −0.035 (SD: 0.053 for placebo, 0.061 for verum)), a one-sided significance level of 5%, and a power of 80%, a sample size of 76 patients (38 per treatment group) was determined using the two sample t-test, considering a drop-out rate of 5.5% and a death rate of 5.5% as seen in LIPCAL-ALS. During the study, it was determined that 5 additional patients had to be recruited, as the drop-out rate was higher than anticipated. Because of the high drop-out rates observed in previous trials with nutritional interventions, this scenario had been anticipated and implemented in the study protocol, allowing these additionally recruited patients to be randomised and masked in the same way without any protocol amendments.

All randomised patients were analysed according to the intention-to-treat principle. To analyse the change of NfL serum levels during the study, we calculated slopes from a univariate linear regression model separately for each patient. Group comparisons of NfL slopes were done using the Wilcoxon rank sum test with a one-sided significance level of 5%. As sensitivity post-hoc analysis, we also performed a one-step linear mixed-effects regression model. For primary analysis, missing data were not replaced. Explorative post-hoc regression analyses for multiple imputation of missing NfL slopes (multivariate imputation by fully conditional specification, FCS) are given with group, age, SVC, NfL, and BMI as influencing factors.

The Wilcoxon rank sum test with a two-sided significance level of 5% was performed for comparing both groups for secondary outcomes. Multiple linear regression models were applied for both primary and secondary outcomes to adjust for potential confounders, including NfL baseline levels, age, BMI, and SVC as the main independent prognostic factors in ALS. These factors were not pre-specified in the protocol and decided on post-hoc. NfL baseline levels were favoured over pre-baseline ALSFRS-R progression rate based on recent publications highlighting their predictive value.^42^ All analyses for secondary outcomes parameters were exploratory and interpreted as hypothesis-generating. As additional calories applied via nutritional supplements were evenly distributed between both groups, this parameter was not included in the models. Normal distributions for NfL and BMI were checked by visual assessment.

For group differences of survival, Kaplan Meier curves and log-rank test were used. Furthermore, subgroup analyses were performed for patients with slow vs. fast disease progression, i.e., patients with NfL baseline values </≥ median as well as for patients in later vs. earlier disease stages, i.e., patients with ALSFRS-R baseline scores </≥ median. Additional analyses were performed for the per-protocol set, consisting of patients who sufficiently adhered to the study protocol (intake of >65% of treatment dosage).

Adverse events are reported by descriptive analysis. Statistical analyses were done using SAS (version 9.4), R (version 4.5.0), and GraphPad Prism (version 10.3.0).

### Role of the funding source

This was an investigator-initiated trial funded by a private donation. The funder of the study had no role in study design, data collection, data analysis, data interpretation, or writing of the report.

## Results

Between April 5, 2022, and February 12, 2025, 81 patients were screened in the Department of Neurology of Ulm University, Ulm, Germany. The trial ended as planned according to protocol. All 81 patients were enrolled and randomly assigned to receive either oral βHB (n = 40) or placebo (n = 41). All patients were included in the full analysis data set (Fig. 1). Dropouts did not differ between verum and placebo regarding baseline characteristics, and dropouts and non-dropouts were similar regarding ALSFRS-R and NfL at baseline as well as ALSFRS-R progression rate pre-baseline, suggesting that dropouts did not significantly bias study results.

**Fig. 1:**
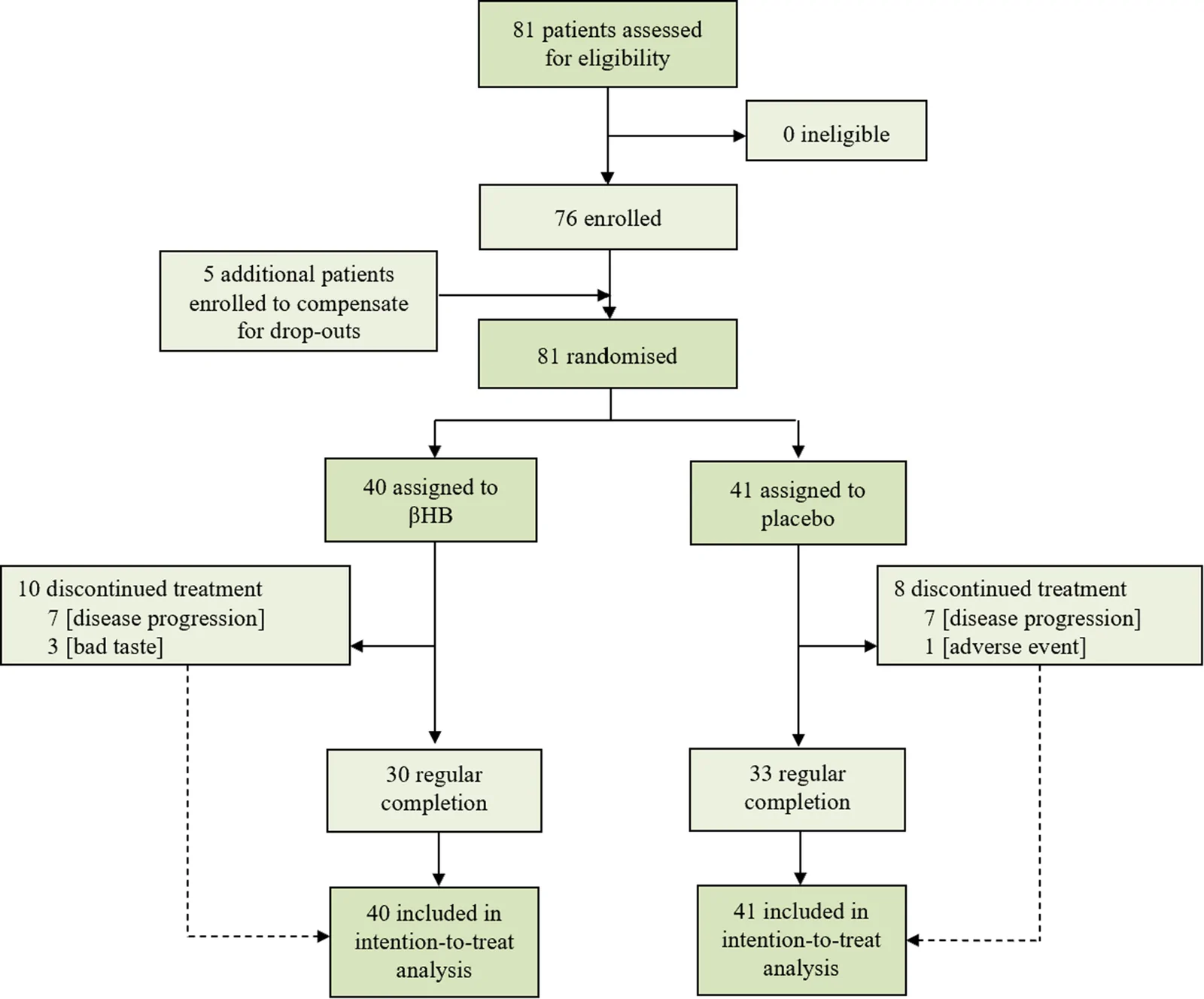
**Trial profile.** βHB: oral D β-hydroxybutyrate ester.

Baseline characteristics are shown in Table 1. Importantly, despite randomisation, βHB patients featured worse SVC and higher NfL levels at baseline compared to placebo, indicating worse overall condition and faster disease progression in the βHB group as NfL levels at baseline showed a positive correlation with disease progression as measured by loss of ALSFRS-R per month during the intervention (Pearson’s correlation coefficient r = 0.46, p = 0.0001; Supplementary Fig. S1). On the other hand, patients in the placebo group were older compared to βHB, representing a potential disadvantage for the placebo group as higher age is a well-known negative prognostic factor. There was a higher share of patients receiving nutritional supplements at baseline in the βHB group compared to placebo, which might have affected the results. All other factors were similar in both groups. Of note, 86.5% of patients showed a resting energy expenditure higher than their calculated value as signified by a metabolic ratio (MR) of >100%, and 70.3% displayed an MR of >110%, commonly referred to as hypermetabolic.

**Table 1:** Patient characteristics at baseline.

	Total	βHB	Placebo
Number of patients	81	40	41
Age (years)	62.0 (52.0–67.0)	59.0 (50.5–66.5)	64.0 (60.0–67.0)
Sex			
Female	39 (48.1%)	19 (47.5%)	20 (48.8%)
Male	42 (51.9%)	21 (52.5%)	21 (51.2%)
Onset			
Spinal	55 (67.9%)	27 (67.5%)	28 (68.3%)
Bulbar	26 (32.1%)	13 (32.5%)	13 (31.7%)
NfL (pg/mL)	97.9 (63.7–152.0)	113.0 (65.9–170.5)	91.0 (63.7–138.0)
ALSFRS-R	33.0 (27.0–37.0)	33.0 (26.0–37.5)	33.0 (27.0–36.0)
BMI (kg/m^2^)	25.3 (21.8–27.8)	25.3 (21.7–27.5)	25.3 (22.1–28.2)
SVC (%)	72.0 (53.0–89.0)	64.5 (41.0–84.0)	79.0 (58.0–96.0)
Weight loss pre-BL (kg)	0.0 (0.0–4.0)	0.0 (0.0–2.5)	0.0 (0.0–4.0)
Yes	34 (42.0%)	17 (42.5%)	17 (41.5%)
No	47 (58.0%)	23 (57.5%)	24 (58.5%)
Progression rate pre-BL	0.66 (0.45–1.00)	0.66 (0.45–0.89)	0.66 (0.46–1.05)
≤0.77 points/month	49 (60.5%)	25 (62.5%)	24 (58.5%)
>0.77 points/month	32 (39.5%)	15 (37.5%)	17 (41.5%)
Disease duration (months)	20.0 (14.0–30.0)	21.0 (13.3–30.8)	20.0 (14.0–29.0)
Patients with high-caloric supplements	25 (30.9%)	16 (40.0%)	9 (22.0%)
Average amount of calories applied by supplements (kcal)	400 (300–750)	400 (300–675)	400 (275–1200)
EQ-5D-5L	50 (40–70)	50 (38–70)	50 (40–75)
CNAQ	28.0 (26.0–30.0)	28.5 (26.0–31.0)	28.0 (25.0–30.0)
mRMR (kcal/day)	1845 (1540–2165)	1807 (1471–2197)	1896 (1589–2067)
Metabolic rate (%)	123 (107–142)	114 (104–144)	125 (112–141)
Respiratory quotient	0.65 (0.54–0.72)	0.66 (0.53–0.72)	0.64 (0.55–0.74)
Fat mass (kg)	23.8 (17.3–31.4)	25.3 (17.2–31.4)	23.2 (17.3–31.6)
Fat free mass (kg)	49.1 (39.7–61.6)	49.7 (40.4–64.4)	47.9 (38.9–61.6)
BCM (kg)	22.7 (17.5–30.2)	23.5 (17.9–30.7)	22.6 (16.6–28.5)
ECM (kg)	26.6 (22.3–32.9)	26.2 (22.5–30.9)	26.7 (22.2–33.3)
ECM/BCM	1.19 (1.08–1.35)	1.15 (1.06–1.35)	1.23 (1.11–1.35)
TBW (L)	36.5 (29.9–45.3)	37.0 (29.5–47.3)	36.5 (29.9–45.3)
ECW (L)	17.3 (14.7–20.1)	17.4 (14.2–20.1)	17.2 (15.1–20.2)
Phase angle (°)	4.2 (3.6–5.2)	4.4 (3.8–5.2)	4.0 (3.4–5.2)
Triglycerides (mmol/L)	1.4 (1.0–1.8)	1.3 (0.9–1.8)	1.5 (1.1–1.9)
Cholesterol (mmol/L)	5.5 (4.6–6.6)	5.3 (4.6–6.3)	5.5 (4.6–6.6)
LDL (mmol/L)	3.2 (2.5–4.1)	3.0 (2.6–4.2)	3.3 (2.4–4.1)
HDL (mmol/L)	1.6 (1.3–1.9)	1.6 (1.3–2.0)	1.5 (1.3–1.9)
Acetone (urine) (mg/L)	1.5 (1.1–2.2)	1.5 (1.0–2.2)	1.4 (1.1–2.3)
β-hydroxybutyrate (μmol/L)	80.0 (40.0–205.6)	74.6 (40.5–223.3)	80.1 (39.9–179.2)
Acetoacetate (μmol/L)	49.4 (26.2–82.9)	51.4 (25.3–95.1)	45.8 (26.9–76.9)
Total ketone bodies (μmol/L)	123.1 (68.2–295.6)	123.1 (63.7–329.9)	122.8 (69.9–263.0)

Data are given as median (interquartile range), n or n (%). Wilcoxon rank sum test or chi square test. Bold p-values signify significant differences. βHB: oral D-β-hydroxybutyrate ester, NfL: neurofilament light chain level in serum, ALSFRS-R: amyotrophic lateral sclerosis functional rating scale revised, BMI: body mass index, SVC: slow vital capacity, CNAQ: Council on Nutrition Appetite Questionnaire, mRMR: measured resting metabolic rate, ECM: Extracellular Mass, BCM: Body Cell Mass; TBW: Total Body Water; ECW: Extracellular Water; LDL: Low-density Lipoprotein; HDL: High-density Lipoprotein; BL: baseline.

Regarding NfL as the primary endpoint of the study, patients in the βHB group showed a decline of NfL levels over the intervention time of 6 months (NfL slope), whereas NfL levels remained stable in the placebo group (−0.5 pg/mL/month (−3.2 to 2.0, minimum −11.2, maximum 10.4) vs. 0.0 pg/mL/month (−1.7 to 3.3, minimum −6.2, maximum 49.0), median difference −1.3 pg/mL/month (95% CI - ∞ to 0.34), p = 0.094, Fig. 2). Individual unadjusted values are presented in Supplementary Fig. S2, which suggested normal distribution based on visual assessment (Supplementary Fig. S3).

**Fig. 2:**
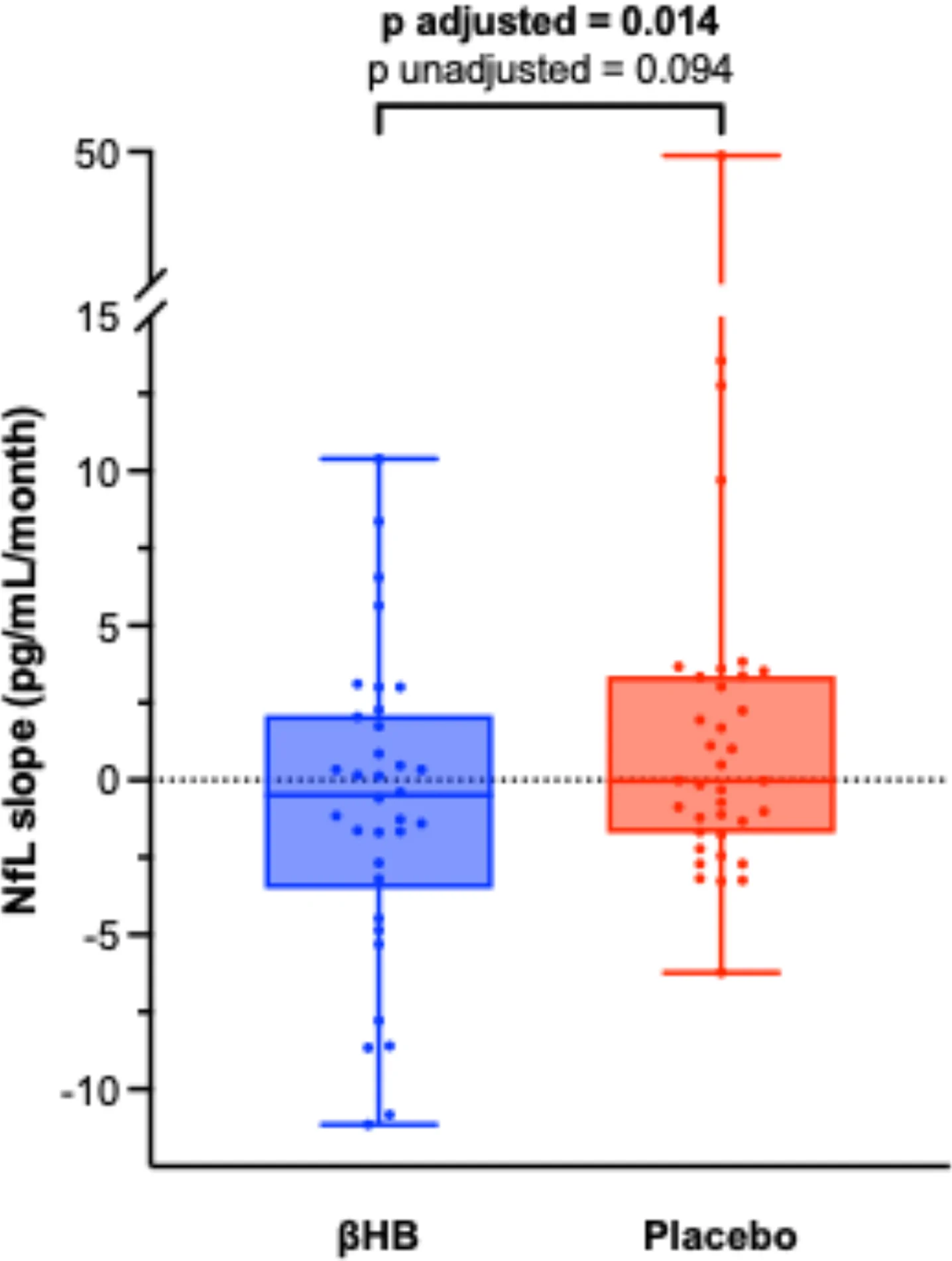
**NfL slope in βHB and placebo.** Box plots show median, IQR, minimum and maximum. Wilcoxon rank sum test. Bold p-values refer to statistically significant values. Adjusted p-values refer to multiple linear regression models with group, age, baseline NfL, BMI, and baseline SVC as influencing variables. βHB n = 34, placebo n = 37. βHB: oral D β-hydroxybutyrate ester, NfL: neurofilament light chain levels in serum.

After adjusting for baseline NfL, age, BMI, and SVC, the group effect was statistically significant (−1.5 pg/ml/month in βHB vs. +2.8 pg/ml/month in placebo, mean difference −4.2 pg/ml/month (95% CI - ∞ to −1.1, p = 0.014, Fig. 2)). For 10 patients with only one available NfL value, individual NfL slopes could not be analysed. Applying a regression model for multiple imputation of missing NfL slopes, the adjusted difference between βHB and placebo remained significant (mean difference −4.3 pg/mL/month, 95% CI -∞ to −1.1, p = 0.013). A similar effect was observed when analysing the per protocol set comprising patients who adequately adhered to the study protocol, i.e., patients with a cumulative intake of at least 65% of treatment dosage (n = 59, mean difference −3.8 pg/mL/month, 95% CI −7.6 to 0.1, p = 0.058; Supplementary Table S2). Applying the one-step linear mixed-effects model as a sensitivity analysis, the effect in the intention-to-treat cohort was not significant (−0.4 pg/ml/month in βHB vs. +0.3 pg/ml/month in placebo, mean difference −0.7 pg/mL/month, 95% CI -∞ to 1.5, p = 0.30).

Regarding secondary endpoints, quality of life, as examined by the EQ-5D-5L questionnaire, showed a slower decline in the βHB group compared to placebo (adjusted p = 0.040, Table 2). There was no difference for the remaining secondary outcomes, including clinical parameters such as ALSFRS-R, BMI, and survival (Table 2). Of note, patients in the verum group showed a faster decline of ALSFRS-R (−1.4 points/month (IQR −2.1 to 0.6) vs. −0.8 points/month (IQR −1.2 to −0.2), p = 0.020), which might be contributed to a higher share of patients with faster natural disease progression in the verum group, as indicated by higher NfL baseline levels, although this possible explanation cannot be proven by the study data.

**Table 2:** Primary and secondary Outcomes.

	βHB	Placebo	p-value	Adjusted p-value
NfL (slope) (pg/mL/month)	−0.5 (−3.2 to 2.0)	0.0 (−1.7 to 3.3)	0.094	**0.014**
ALSFRS-R (slope) (pt./month)	−1.4 (−2.1 to −0.6)	−0.8 (−1.2 to −0.2)	**0.020**	0.30
BMI (slope) (kg/m^2^/month)	0.0 (−0.2 to 0.1)	−0.1 (−0.2 to 0.1)	0.66	0.30
SVC (slope) (%/month)	−2.0 (−3.1 to 0.0)	−1.7 (−2.7 to 0.3)	0.44	0.92
MR (slope) (per month)	−0.2 (−1.7 to 2.8)	0.6 (−3.4 to 1.6)	0.65	0.82
RQ (slope) (per month)	0.00 (−0.02 to 0.01)	0.00 (−0.02 to 0.01)	0.38	0.23
Phase angle (slope) (°/month)	−0.1 (−0.2 to −0.1)	−0.1 (−0.1 to 0.0)	0.052	0.33
FM (slope) (kg/month)	0.4 (−0.3 to 1.1)	0.3 (−0.1 to 0.8)	0.75	0.19
ECM/BCM (slope) (per month)	0.02 (0.00 to 0.04)	0.01 (−0.00 to 0.02)	0.56	0.65
CNAQ (slope) (pt./month)	−0.2 (−0.6 to 0.1)	−0.3 (−0.9 to 0.1)	0.33	0.22
EQ-5D-5L (slope) (pt./month)	−1.7 (−3.4 to 0.6)	−1.7 (−5.3 to 0.0)	0.34	**0.040**
Triglycerides (slope) (mmol/L/month)	0.02 (−0.03 to 0.05)	0.00 (−0.10 to 0.07)	0.38	0.27
Cholesterol (slope) (mmol/L/month)	−0.05 (−0.13 to 0.03)	0.02 (−0.14 to 0.07)	0.37	0.65
LDL/HDL (slope) (per month)	0.01 (−0.05 to 0.06)	0.01 (−0.04 to 0.07)	0.95	0.93
Total ketone bodies (slope) (μmol/L/month)	8.15 (−1.98 to 35.84)	4.10 (−11.07 to 26.48)	0.42	0.26
βHB (slope) (μmol/L/month)	4.72 (−2.53 to 26.09)	3.46 (−7.07 to 20.95)	0.50	0.24
Acetoacetate (slope) (μmol/L/month)	2.60 (−0.65 to 11.47)	0.70 (−4.16 to 6.65)	0.25	0.32
Acetone (slope) (mg/L/month)	0.13 (−0.08 to 1.05)	0.06 (−0.11 to 0.34)	0.30	0.45
Death/tracheostomy	2/40 (5.0%)	4/41 (9.8%)	0.55	–

Wilcoxon rank sum test. Log-rank test for survival. Values signify median slopes (change per month). Bold p-values signify significant differences. Adjusted p-values refer to multiple linear regression models with group, age, baseline NfL, baseline BMI, and baseline SVC as well as baseline values of respective variables as influencing variables. p-values refer to one-sided testing for NfL slope (primary endpoint) and two-sided testing for all secondary outcomes. βHB: oral D-β-hydroxybutyrate ester, NfL: neurofilament light chain level in serum, ALSFRS-R: amyotrophic lateral sclerosis functional rating scale revised, BMI: body mass index, SVC: slow vital capacity, MR: metabolic ratio, RQ: respiratory quotient, FM: fat mass, ECM/BCM: extracellular mass/body cell mass, CNAQ: Council of Nutrition Appetit Questionnaire, EQ-5D-5L: life quality questionnaire.

The positive effect of βHB on NfL slope was more pronounced in patients with higher NfL levels (≥median) at baseline. In this subgroup, median NfL levels increased in the placebo group, whereas they decreased in the βHB group (+1.7 pg/mL/month (−1.3 to 9.7) vs. −1.3 pg/mL/month (−5.9 to 3.0), adjusted p = 0.045 (Fig. 3)).

**Fig. 3:**
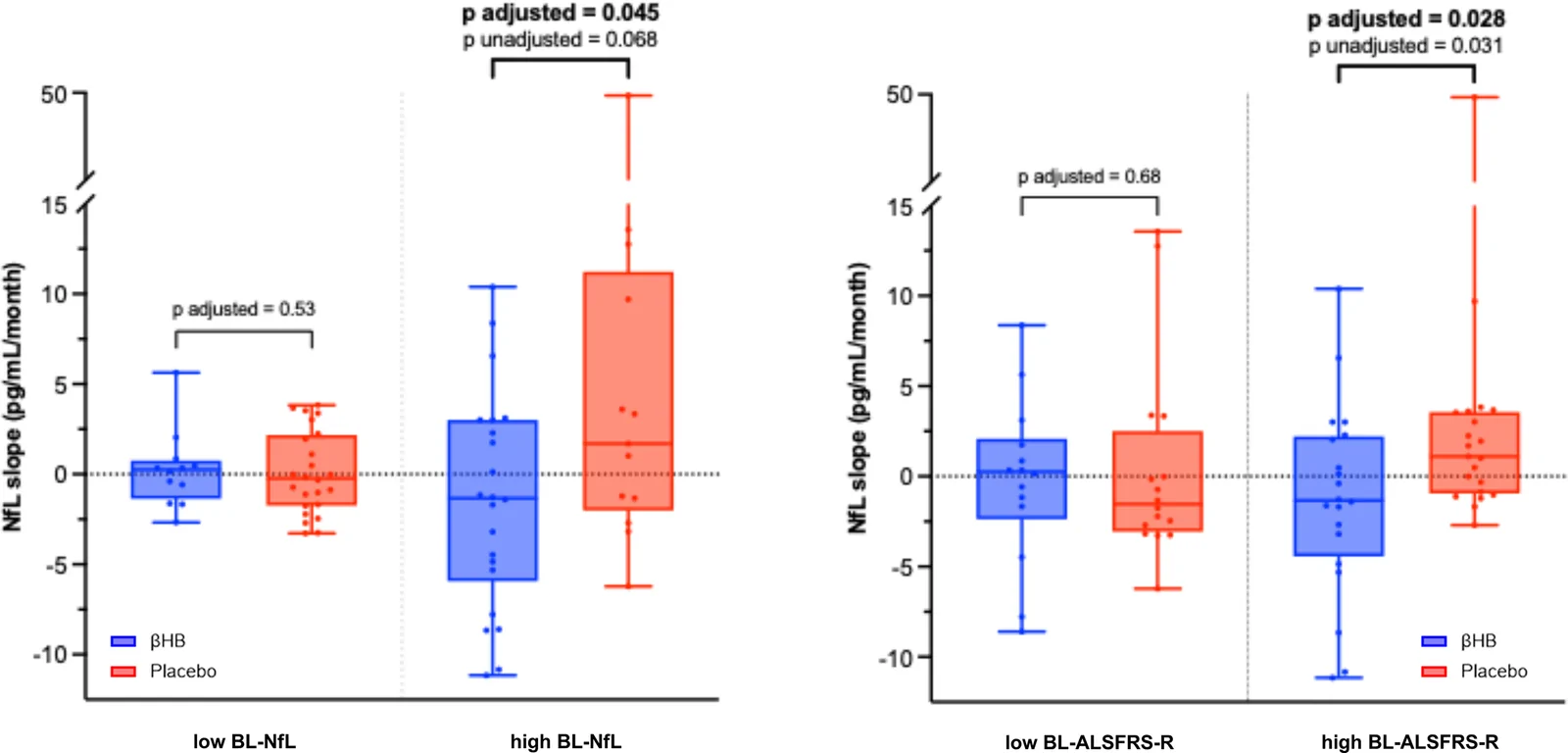
**NfL slope for baseline NfL and ALSFRS-R subgroups.** Box plots show median, IQR, minimum and maximum. Wilcoxon rank sum test. Bold p-values refer to statistically significant values. Adjusted p-values refer to multiple linear regression models with group, age, baseline NfL, BMI, and baseline SVC as influencing variables. For the ALSFRS-R subgroup, baseline ALSFRS-R was used as an additional influencing variable. Subgroups “low and “high” refer to <median vs. ≥median. For low BL-NfL: βHB n = 12, placebo n = 24. For high BL-NfL: βHB = 22, placebo = 13. For low BL-ALSFRS-R: βHB n = 14, placebo n = 16. For high BL-ALSFRS-R: βHB n = 20, placebo n = 21. βHB: oral D β-hydroxybutyrate ester, NfL: neurofilament light chain levels in serum, ALSFRS-R: amyotrophic lateral sclerosis functional rating scale revised, BL: baseline.

Moreover, patients with higher ALSFRS-R values (≥median) at baseline showed an increase of median NfL levels per month in placebo vs. a median decrease of NfL levels per month in the βHB group (+1.1 pg/mL/month (−0.9 to 3.5) vs. −1.3 pg/mL/month (−4.0 to 2.2), adjusted p = 0.028 (Fig. 3)). ALSFRS-R baseline values (sum scores) were negatively correlated with disease duration (Pearson’s correlation coefficient r = −0.46, p < 0.0001, Supplementary Fig. S1).

Regarding urinary acetone levels, post-hoc exploratory analyses indicated that the verum group exhibited an increase during the first half of the study, followed by a decrease at the end of study (1.5 mg/L (1.0 to 2.2) at baseline, 6.9 mg/L (1.7 to 92.2) after 3 months, and 2.7 mg/L (1.3 to 9.5), after 6 months, Supplementary Table S3). This pattern was also evident in most individual patients (Supplementary Fig. S4). In contrast, as expected, acetone levels were relatively stable in the control group. The course of serum βHB showed a similar pattern compared to acetone, although the effects were less pronounced (Supplementary Table S3). Verum patients with higher acetone increases (≥median) after 3 months had better NfL slopes compared to placebo (−1.7 pg/mL/month (−5.3 to 0.3) vs. 0.0 pg/mL/month (−1.7 to 3.4), p = 0.014) (Fig. 4). In contrast, patients with lower acetone levels (<median), showed relatively stable NfL slopes (0.1 pg/mL/month (−1.7 to 3.0)), which were similar to placebo (p = 0.95). Multiple regression analyses for secondary endpoints are given in Supplementary Tables S4–S17.

**Fig. 4:**
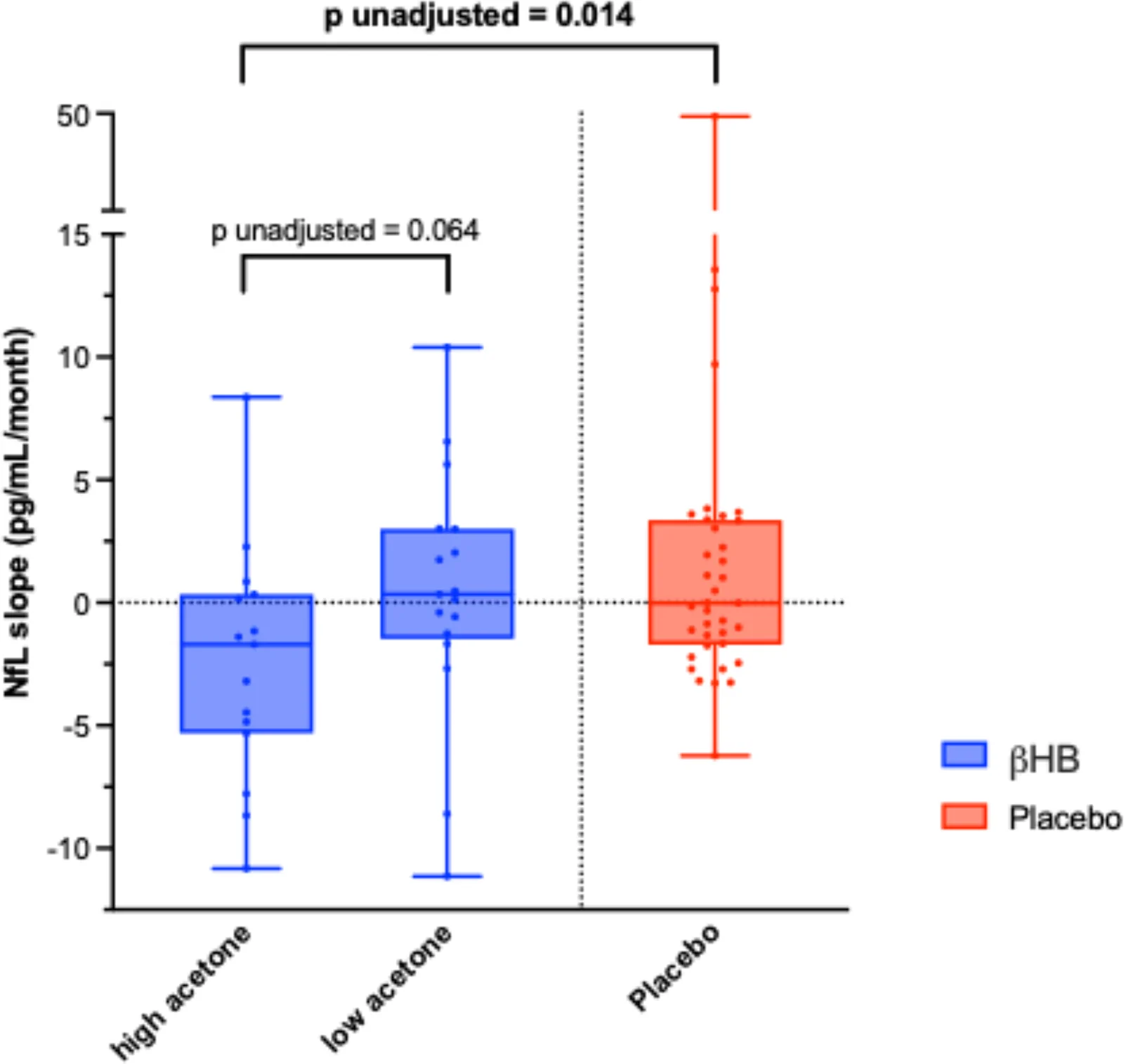
**Effect of acetone levels on NfL.** Boxplots show NfL slopes (median, IQR, minimum, and maximum) in patients with high (n = 15) and low acetone levels (n = 17) after 3 months of βHB treatment compared to placebo (n = 37). Wilcoxon rank sum test. Bold p-values refer to statistically significant values. Subgroups “high” and “low” refer to ≥ median vs. < median. βHB: oral D β-hydroxybutyrate ester, NfL: neurofilament light chain levels in serum, BL: baseline.

βHB was well tolerated as adverse events were mainly mild gastrointestinal symptoms or unpleasant feeling in the throat after intake (Table 3). There were no significant differences between βHB and placebo except for nausea/vomiting, which was more frequent in the placebo group (29.3% vs. 7.5%). We did not record any intervention-related serious adverse events or any concerning laboratory abnormalities.

**Table 3:** Safety analysis.

	βHB n = 40	Placebo n = 41
Nausea/vomiting	3 (7.5%)	12 (29.3%)
Increased stool frequency/diarrhoea/decreased stool consistency	8 (20.0%)	6 (14.6%)
Obstipation	1 (2.5%)	1 (2.5%)
Burning throat after intake	8 (20.0%)	3 (7.3%)
Abdominal pain/heartburn	5 (12.5%)	8 (19.5%)
Feeling of fullness	0 (0.0%)	1 (2.4%)
Feeling of hunger after intake	0 (0.0%)	1 (2.4%)
AE	25 (62.5%)	32 (78.0%)
SAE	0 (0.0%)	0 (0.0%)

Data are given as n (%). AE: Adverse Event, SAE: Serious Adverse Event, βHB: oral D-β-hydroxybutyrate ester. Only intervention-related SAEs are given.

In total, there were 18 dropouts (22.2%), 10 in the βHB (25.0%) and 8 in the placebo group (19.5%). Bad taste and an increased burden associated with study visits due to clinical worsening were identified as the most common reasons for dropouts. Only one patient in the placebo group terminated the study due to gastrointestinal symptoms. Nausea and vomiting were more frequent in the placebo group (29.3% vs. 7.5%), possibly due to the placebo formulation containing a high share of glucose (40.0%). Overall, 97.0% (75.0%–100.0%) of study intervention/placebo was consumed during active study participation of each patient, signifying excellent study adherence. Comparing both study groups, compliance was similar in both groups with a slight tendency towards higher compliance in the placebo group (98.0% (90.2–99.3) vs. 92.3% (67.5–99.6), p = 0.15). The nutrition and appetite questionnaires (CNAQ and UNQ) revealed both increased (5.7% βHB, 5.3% placebo) and reduced appetite (42.9% βHB, 42.1% placebo) as well as both increased (2.9% βHB, 2.6% placebo) and reduced food intake (11.4% βHB, 26.3% placebo) in both groups. The total CNAQ score remained stable in the βHB group, whereas it decreased in the placebo group (0 points (−3 to 1) vs. −2 points (−7 to 1), p = 0.19). However, there were no significant differences in both scores.

## Discussion

In this randomised, placebo-controlled study, we investigated efficacy and tolerability of oral ketone bodies in ALS, aiming at alleviating the energy deficit. We focused on NfL as biological biomarker, since recent studies have shown that clinical parameters such as ALSFRS-R and survival are unlikely to show a significant effect within an intervention period of 6 months.^19^, ^43^ On the other hand, NfL has meanwhile been established as a reliable prognostic biomarker, signifying axonal damage and correlating strongly with disease progression and survival,^42^ while also responding earlier compared to other clinical endpoints.^20^, ^43^ Therefore, this study aimed at evaluating efficacy on a biological level based on NfL before conducting a subsequent follow-up study with larger sample size and longer duration to evaluate clinical endpoints.

Regarding baseline characteristics, we found that most study participants (about 70%) were hypermetabolic at baseline. Of note, despite randomisation, patients in the βHB group had lower SVC and higher NfL baseline levels, indicating that those patients were in further advanced disease stages as well as experiencing faster disease progression, although this imbalance might have been partly counteracted by a higher median age in the placebo group. We found that NfL serum levels as the primary endpoint, measured as slope over six months, showed a decline in the βHB group, but were stable in placebo, indicating a potential beneficial effect of the intervention. While this difference was not statistically significant in the unadjusted analysis, it was significant after applying multiple linear regression models to account for baseline NfL, age, BMI, and SVC. Similar trends were observed in the per protocol analyses, which only included patients sufficiently adhering to the study protocol. However, since these effects were only evident after adjustment and statistical significance was lost in the one-step linear mixed-effects model applied as sensitivity analysis, they must be confirmed, together with respective functional outcome measures, in a follow-up trial with longer treatment duration.

Although the observed NfL differences between verum and placebo may appear not very prominent at first glance, it has to be noted that these effects relate to a relatively short time frame, and that even small NfL changes may be followed by significant clinical implications long-term similar to the tofersen and LIPCAL studies, which showed positive signals for NfL prior to clinical outcomes.^19^, ^20^, ^43^

In exploratory analyses, patients receiving βHB typically showed an increase in urinary acetone levels, which reflect the metabolic turnover of βHB over several hours. Peak acetone levels were reached during the first half of the study, followed by a decrease. After a few weeks on ketogenic diet or during starvation, this phenomenon occurs as utilisation of ketone bodies becomes more efficient due to higher renal reabsorption rates and is known as “keto-adaption”.^44^, ^45^ The similar pattern of acetone in our study may therefore indicate a metabolic adaption to the additional, novel form of energy substrates. In terms of acetone peak levels, we observed large individual differences, which may relate to compliance or individual differences in metabolisation. Of note, patients with higher acetone increases showed significantly better NfL slopes compared to placebo. However, this analysis was conducted post-hoc and is limited by the small sample sizes of subgroups. As opposed to acetone, serum βHB strongly depends on the time interval between intake of βHB and blood sampling, since exogenous βHB only prompts temporary elevations rather than continuously stable levels.^31^ Therefore, single measurements of βHB serum levels appear to be less suitable for monitoring the metabolisation of oral ketone bodies and, consistently, showed a similar, but overall weaker association with NfL in this study. As longitudinal adherence data were not available, it cannot be ruled out completely that decreasing acetone levels during the study were caused by declining compliance; however, given the overall high compliance rates of >90% in the verum group, this seems unlikely.

Prespecified subgroup analysis revealed that the effect on NfL might be more pronounced in fast progressors and patients with better overall functional status, mirroring the results of a former trial with a high-caloric, fat-rich intervention.^19^, ^20^ These findings must be interpreted as hypotheses-generating as the study was not sufficiently powered for subgroup analyses. With this limitation in mind, the results might indicate that high-energetic substrates might be more effective at an early stage to provide additional energy to counteract the energy deficit. These hypotheses are in line with a recent study, which showed that ALS patients mobilise additional energy sources in form of fatty acids and ketone bodies already in early disease stages.^25^ However, since these assumptions are based on small subgroups, they must be verified by subsequent studies.

As opposed to NfL, we could not detect any significant effects on BMI, ALSFRS-R and survival. In particular, the faster ALSFRS-R decline in the verum group in the unadjusted analysis raises concerns and seemingly contradicts the beneficial NfL findings, although this finding lost statistical significance in the adjusted analysis. Of note, this proof-of-concept study was neither designed not powered to capture clinical effects, but rather to provide evidence for efficacy on a biological level. Consequently, a subsequent larger study with higher statistical power and longer treatment duration should be conducted to verify the effects on NfL and to clarify, whether βHB provides meaningful clinical effects. Based on the lessons learned from this study, a follow-up trial should feature a substantially higher sample size to account for the relatively small NfL effect observed here as well as a significantly longer duration to evaluate clinical endpoints. Prospective preparational studies might help to study the natural trajectory of metabolic variables such as metabolic rate and BIA parameters in ALS and to decide, whether such parameters should be considered as inclusion/exclusion criteria or stratification factors.

Overall, we found that βHB was well tolerated. While nausea and vomiting were more frequent in the placebo group, other clinically relevant side effects were about evenly distributed. We did not register any treatment-related serious adverse events. However, βHB patients overall showed a slightly worse compliance and a higher drop-out rate compared to placebo, which was mainly caused by the bad taste of the intervention. Therefore, future trials should also focus on improving the taste of oral ketone bodies. Moreover, the sample size of this study might be too small to detect all possible side effects, and the study duration might be too short to capture longer-term tolerability issues.

## Contributors

Conceptualisation: CH, JDo; Data Curation: CH, JDo, JDr, TH, FB; Formal Analysis: CH, JDo, JDr, BM; Investigation: CH, LJ, TH, JDo, UW, ÖP, TR, FW; Methodology: CH, JDo, JDr, BM, HT; Project Administration: CH, JDo, JS, LJ; Resources: CH, JDo; Supervision: CH, JDo, JS, HT; Visualisation: JDo, CH, JDr, BM; Writing - original draft: CH, JDo; Writing - review and editing: CH, JDo, LJ, ZE, TH, EF, FB, TR, FW, ÖP, UW, FB, LR, MW, ZU, SB, SW, HT, JHW, BM, LM, JDr, JS. CH and JDo have directly accessed and verified the underlying data reported in the manuscript. All authors confirm that they read and approved the final version of the manuscript.

## Data sharing statement

Individual participant data that underlie the results reported in this article, after de-identification (text, tables, and figures) will be available. Data will be available beginning 3 months and ending 5 years following article publication. Data will be shared with researchers who provide a methodologically sound proposal. Data will be shared for analyses to achieve the aims in the approved proposal. Proposals should be directed to johannes.dorst@uni-ulm.de; to gain access, data requestors will need to sign a data access agreement. Data are available for 5 years at https://www.uniklinik-ulm.de/neurologie.html.

## Declaration of interests

JDo reports research grants and personal fees from Nutricia GmbH, outside the submitted work. All other authors declare that they have no conflict of interest.

## Appendix A Supplementary data

Supplementary Tables S1–S17

KETO-ALS Study Protocol
