# Supplementary material for: Efficacy and tolerability of D-β-hydroxybutyrate ester in patients with amyotrophic lateral sclerosis (KETO-ALS)–a randomised, placebo-controlled, double-blind study
Source: eClinicalMedicine. 2026 Oct 26;101:104272. doi: 10.1016/j.eclinm.2026.104272 (PMC13734945; doi:10.1016/j.eclinm.2026.104272)
Supplement: Supplementary file 1 [file mmc1.pdf]

Supplement

Efficacy and tolerability of D-β-hydroxybutyrate ester in patients with amyotrophic lateral sclerosis (KETO-ALS) - a randomised, placebo-controlled, double-blind study

Christine Herrmann[1], Luisa Jagodzinski[1], Zeynep Elmas[1], Tabea Herschmann[1], Elke Fröhlich[1], Franziska Bachhuber[1], Tanja Ruschitzka[1], Frank Willkomm[1], Özlem Parlak[1], Ulrike Weiland[1], Felicitas Becker[1], Lars Richter[1], Maximilian Wiesenfarth[1], Zeljko Uzelac[1], Sebastian Michels[1], Simon Witzel[1], Hayrettin Tumani[1], Jochen H. Weishaupt[1,2], Ludwig Maier[4], Benjamin Mayer[3], Jens Dreyhaupt[3], Joachim Schuster[1,2], Johannes Dorst[1]

[1] Department of Neurology, University of Ulm, Ulm, Germany

[2] German Center for Neurodegenerative Diseases (DZNE), Ulm, Germany

[3] Institute of Epidemiology and Medical Biometry, University of Ulm, Ulm, Germany

[4] Ulm University Hospital pharmacy, University of Ulm, Ulm, Germany

Table of Contents:

Supplementary Table 1: Content of βHB and placebo page 3

Supplementary Table 2: Per protocol analysis page 4

Supplementary Table 3: Parameters of ketone metabolism page 5

Supplementary Tables 4-17: Multiple regression analyses pages 6-10

Ulm Nutrition Questionnaire pages 11-12

Tables

Suppl. Table 1: Content of βHB (KE4®, KetoneAid, Falls Church, USA) and Placebo per dosage (20 mL)
βHB: oral D-beta-hydroxybutyrate ester

	βHB	Placebo
**Ingredients**	βHB<br>Water<br>Citric Acid<br>Allulose<br>Organic Stevia<br>Natural Flavors<br>Potassium Sorbate	Glucose Solution 40%<br>Bitter Orange Peel Tincture (3 drops/200 mL)<br>Ginger Tincture (3 drops/200 mL)
**Calories (kcal)**	48	32
**Fat**	0%	0%
**Cholesterol**	0%	0%
**Sodium**	12 mg	5.6 mg
**Carbohydrates**	167 mg	8.0 g
**Protein**	0%	0%
**Potassium**	33 mg	-
**pH**	4.6	4.0

Suppl. Table 2: Per protocol analysis: Primary and secondary Outcomes

Wilcoxon rank sum test. Log-rank test for survival. Values signify median slopes (change per month). Bold p-values signify significant differences. Adjusted p-values refer to multiple linear regression models with group, age, baseline NfL, baseline BMI, and baseline SVC as well as baseline values of respective variables as influencing variables. βHB: oral D-β-hydroxybutyrate ester, NfL: neurofilament light chain level in serum, ALSFRS-R: amyotrophic lateral sclerosis functional rating scale revised, BMI: body mass index, SVC: slow vital capacity, MR: metabolic ratio, RQ: respiratory quotient, FM: fat mass, ECM/BCM: extracellular mass/body cell mass, CNAQ: Council of Nutrition Appetit Questionnaire, EQ-5D-5L: life quality questionnaire

	βHB	Placebo	p-value	Adjusted p-value
**NfL (slope)** (pg/mL/month)	-0·1 (-1·7 to 2·0) n=30	-0·2 (-1·8 to 3·0) n=35	0·55 (favouring placebo)	**0·058** (favouring βHB)
**ALSFRS-R (slope)** (pt./month)	-1·2 (-2·1 to -0·8) n=30	-0·8 (-1·2 to -0·3) n=36	**0·055**	0·74
**BMI (slope)** (kg/m^2/month)	-0·05 (-0·22 to 0·16) n=30	-0·04 (-0·14 to 0·13) n=36	0·91	0·24
**SVC (slope)** (%/month)	-1·7 (-3·1 to 4·8) n=30	-1·4 (-2·7 to 0·3) n=34	0·64	0·86
**MR (slope)** (per month)	-0·2 (-2·3 to 2·5) n=28	-0·3 (-3·4 to 1·5) n=33	0·90	0·85
**RQ (slope)** (per month)	0·002 (-0·008 to 0·005) n=28	-0·003 (-0·016 to 0·007) n=33	0·32	0·26
**Phase angle (slope)** (°/month)	-0·12 (-0·17 to -0·05) n=28	-0·07 (-0·13 to -0·03) n=33	0·094	0·31
**FM (slope)** (kg/month)	0·5 (0·0 to 1·3) n=28	0·4 (0·0 to 0·8) n=33	0·46	0·086
**ECM/BCM (slope)** (per month)	0·01 (0·00 to 0·03) n=28	0·01 (0·00 to 0·02) n=33	0·95	0·25
**CNAQ (slope)** (pt./month)	-0·1 (-0·3 to 0·1) n=30	-0·3 (-0·8 to 0·1) n=35	0·38	0·10
**EQ-5D-5L (slope)** (pt./month)	-1·6 (-3·4 to 0·6) n=30	-1·7 (-5·2 to 0·0) n=36	0·24	**0·019**
**Triglycerides (slope)** (mmol/L/month)	0·02 (-0·02 to 0·05) n=30	-0·03 (-0·11 to 0·07) n=35	0·14	0·14
**Cholesterol (slope)** (mmol/L/month)	-0·04 (-0·11 to 0·03) n=30	0·02 (-0·14 to 0·07) n=35	0·70	0·98
**LDL/HDL (slope)** (per month)	0·02 (-0·03 to 0·07) n=30	0·00 (-0·05 to 0·08) n=35	0·65	0·89
**Total ketone bodies (slope)** (µmol/L/month)	6·67 (-1·98 to 35·84) n=30	4·10 (-11·04 to 25·37) n=36	0·62	0·19
**βHB (slope)** (µmol/L/month)	3·11 (-2·53 to 26·09) n=30	3·46 (-6·99 to 19·46) n=36	0·69	0·18
**Acetoacetate (slope)** (µmol/L/month)	1·92 (-2·28 to 10·91) n=30	0·70 (-3·77 to 6·27) n=36	0·47	0·24
**Acetone (slope)** (mg/L/month)	0·09 (-0·16 to 0·64) n=28	0·06 (-0·10 to 0·33) n=34	0·58	0·92
**Death/tracheostomy**	1/30 (0·03%)	2/36 (0·06%)	0·75	··

Suppl. Table 3: **Parameters of ketone metabolism over the course of the study**

Data are given as median (interquartile range). Wilcoxon rank sum test. Bold p-values signify significant differences between βHB and placebo. βHB: oral D-β-hydroxybutyrate ester; BL: baseline.

	Total	βHB	Placebo	p-value
**Acetone in urine (mg/L)**				
**BL**	1·5 (1·1-2·2)	1·5 (1·0-2·2)	1·4 (1·1-2·3)	0·90
**After 3 months**	2·0 (1·1-6·8)	6·9 (1·7-92·2)	1·3 (0·9-2·0)	**<0·0001**
**After 6 months**	1·9 (1·3-3·8)	2·7 (1·3-9·5)	1·7 (1·1-3·0)	0·14
**β-hydroxybutyrate in serum (µmol/L)**				
**BL**	80·0 (40·0-205·6)	74·6 (40·5-223·3)	80·1 (39·9-179·2)	0·61
**After 3 months**	78·3 (32·6-266·5)	196·1 (49·3-497·3)	59·7 (27·8-161·2)	**0·014**
**After 6 months**	124·8 (35·5-290·8)	129·9 (36·1-349·0)	118·5 (34·6-204·4)	0·46
**Acetoacetate in serum (µmol/L)**				
**BL**	49·4 (26·2-82·9)	51·4 (25·3-95·1)	45·8 (26·9-76·9)	0·96
**After 3 months**	48·2 (31·1-115·9)	93·0 (40·8-188·2)	35·6 (23·4-80·4)	**0·0097**
**After 6 months**	54·6 (30·9-112·1)	57·1 (31·2-120·6)	51·5 (22·1-101·5)	0·45
**Total ketone bodies in serum (µmol/L)**				
**BL**	123·1 (68·2-295·6)	123·1 (63·7-329·9)	122·8 (69·9-263·0)	0·74
**After 3 months**	118·9 (63·3-380·4)	301·7 (90·3-653·4)	95·4 (56·3-245·7)	**0·0096**
**After 6 months**	177·8 (66·4-400·2)	190·1 (75·6-469·6)	165·5 (65·8-323·6)	0·53

Suppl. Table 4: Results of multiple regression analysis for ALSFRS-R-R slope models with group, age, baseline NfL, baseline SVC, baseline BMI and baseline ALSFRS-R-R as covariates.
Estimate: regression coefficient; Lower, Upper: lower resp. upper 95% confidence limit for regression coefficient. Pr > |t|: p-value; R-square: 0.27. βHB: oral D-β-hydroxybutyrate ester, SVC: slow vital capacity, NfL: neurofilament light chain, BMI: body mass index, ALSFRS-R: amyotrophic lateral sclerosis functional rating scale revised, BL: baseline

Effect	Treatment	Estimate	Pr > |t|	Lower	Upper
**Intercept**		-0.2849	0.8251	-2.8457	2.2759
**Group**	Group A: βHB	-0.2817	0.2972	-0.8164	0.2530
**Group**	Group B: Placebo	0	.	.	.
**BL Age**		0.01891	0.1424	-0.00650	0.04433
**BL SVC**		0.005403	0.4162	-0.00777	0.01857
**BL NfL**		-0.00858	0.0004	-0.01323	-0.00393
**BL ALSFRS**		-0.03232	0.1662	-0.07838	0.01374
**BL BMI**		-0.01401	0.5769	-0.06381	0.03580

Suppl. Table 5: Results of multiple regression analysis for BMI slope models with group, age, baseline NfL, baseline SVC and baseline BMI as covariates.
Estimate: regression coefficient; Lower, Upper: lower resp. upper 95% confidence limit for regression coefficient. Pr > |t|: p-value; R-Square: 0.08. βHB: oral D-β-hydroxybutyrate ester, SVC: slow vital capacity, NfL: neurofilament light chain, BMI: body mass index, BL: baseline

Effect	Treatment	Estimate	Pr > |t|	Lower	Upper
**Intercept**		1.1216	0.0776	-0.1274	2.3705
**Group**	Group A: βHB	0.1373	0.3029	-0.1264	0.4009
**Group**	Group B: Placebo	0	.	.	.
**BL Age**		-0.00764	0.2197	-0.01994	0.004659
**BL SVC**		0.000081	0.9764	-0.00533	0.005487
**BL NfL**		-0.00155	0.1827	-0.00384	0.000744
**BL BMI**		-0.02240	0.0742	-0.04704	0.002245

Suppl. Table 6: Results of multiple regression analysis for SVC slope models with group, age, baseline NfL, baseline BMI and baseline SVC as covariates.
Estimate: regression coefficient; Lower, Upper: lower resp. upper 95% confidence limit for regression coefficient. Pr > |t|: p-value; R-Square: 0.18. βHB: oral D-β-hydroxybutyrate ester, SVC: slow vital capacity, NfL: neurofilament light chain, BMI: body mass index, BL: baseline

Effect	Treatment	Estimate	Pr > |t|	Lower	Upper
**Intercept**		3.6510	0.3059	-3.4161	10.7182
**Group**	Group A: βHB	-0.07199	0.9216	-1.5274	1.3834
**Group**	Group B: Placebo	0	.	.	.
**BL Age**		-0.00018	0.9958	-0.06948	0.06912
**BL SVC**		-0.02922	0.0549	-0.05908	0.000636
**BL NfL**		-0.02057	0.0014	-0.03291	-0.00823
**BL BMI**		-0.03129	0.6393	-0.1640	0.1014

Suppl. Table 7: Results of multiple regression analysis for MR slope models with group, age, baseline NfL, baseline BMI, baseline SVC and baseline MR as covariates.
Estimate: regression coefficient; Lower, Upper: lower resp. upper 95% confidence limit for regression coefficient. Pr > |t|: p-value; R-Square: 0.29. βHB: oral D-β-hydroxybutyrate ester, SVC: slow vital capacity, NfL: neurofilament light chain, BMI: body mass index, MR: metabolic rate, BL: baseline

Effect	Treatment	Estimate	Pr > |t|	Lower	Upper
**Intercept**		12.2546	0.0772	-1.3840	25.8933
**Group**	Group A: βHB	-0.2956	0.8165	-2.8360	2.2448
**Group**	Group B: Placebo	0	.	.	.
**BL Age**		0.01728	0.7797	-0.1060	0.1405
**BL SVC**		-0.01441	0.5951	-0.06843	0.03961
**BL NfL**		0.01314	0.2284	-0.00848	0.03477
**BL MR**		-0.09768	0.0005	-0.1502	-0.04519
**BL BMI**		-0.04762	0.6736	-0.2730	0.1777

Suppl. Table 8: Results of multiple regression analysis for RQ slope models with group, age, baseline NfL, baseline BMI, baseline SVC and baseline RQ as covariates.
Estimate: regression coefficient; Lower, Upper: lower resp. upper 95% confidence limit for regression coefficient. Pr > |t|: p-value; R-Square: 0.39. βHB: oral D-β-hydroxybutyrate ester, SVC: slow vital capacity, NfL: neurofilament light chain, BMI: body mass index, RQ: respiratory quotient, BL: baseline

Effect	Treatment	Estimate	Pr > |t|	Lower	Upper
**Intercept**		0.06591	0.0254	0.008409	0.1234
**Group**	Group A: βHB	0.006498	0.2306	-0.00424	0.01724
**Group**	Group B: Placebo	0	.	.	.
**BL Age**		-0.00013	0.6109	-0.00064	0.000382
**BL SVC**		-0.00008	0.4803	-0.00030	0.000144
**BL NfL**		-0.00003	0.5820	-0.00012	0.000066
**BL RQ**		-0.09953	<.0001	-0.1418	-0.05728
**BL BMI**		0.000356	0.4497	-0.00058	0.001292

Suppl. Table 9: Results of multiple regression analysis for phase angle slope models with group, age, baseline NfL, baseline BMI, baseline SVC and baseline phase angle as covariates.
Estimate: regression coefficient; Lower, Upper: lower resp. upper 95% confidence limit for regression coefficient. Pr > |t|: p-value; R-Square: 0.41. βHB: oral D-β-hydroxybutyrate ester, SVC: slow vital capacity, NfL: neurofilament light chain, BMI: body mass index, BL: baseline

Effect	Treatment	Estimate	Pr > |t|	Lower	Upper
**Intercept**		0.08271	0.6682	-0.3014	0.4668
**Group**	Group A: βHB	-0.03864	0.3256	-0.1166	0.03934
**Group**	Group B: Placebo	0	.	.	.
**BL Age**		0.002835	0.1201	-0.00076	0.006430
**BL SVC**		0.000373	0.6560	-0.00129	0.002040
**BL NfL**		-0.00040	0.2227	-0.00105	0.000248
**BL phase angle**		-0.07791	<.0001	-0.1068	-0.04900
**BL BMI**		0.000847	0.8152	-0.00637	0.008064

Suppl. Table 10: Results of multiple regression analysis for FM slope models with group, age, baseline NfL, baseline BMI, baseline SVC and baseline FM as covariates.

Estimate: regression coefficient; Lower, Upper: lower resp. upper 95% confidence limit for regression coefficient. Pr > |t|: p-value; R-Square: 0.14. βHB: oral D-β-hydroxybutyrate ester, SVC: slow vital capacity, NfL: neurofilament light chain, BMI: body mass index, FM: fat mass, BL: baseline

Effect	Treatment	Estimate	Pr > |t|	Lower	Upper
**Intercept**		1.1260	0.3862	-1.4544	3.7064
**Group**	Group A: βHB	0.3441	0.1889	-0.1738	0.8619
**Group**	Group B: Placebo	0	.	.	.
**BL Age**		-0.01077	0.3898	-0.03563	0.01409
**BL SVC**		0.006687	0.2398	-0.00458	0.01795
**BL NfL**		-0.00248	0.2615	-0.00685	0.001894
**BL FM**		-0.03494	0.0751	-0.07353	0.003650
**BL BMI**		0.01596	0.6888	-0.06337	0.09529

Suppl. Table 11: Results of multiple regression analysis for ECM/BCM slope models with group, age, baseline NfL, baseline BMI, baseline SVC and baseline ECM/BCM as covariates.

Estimate: regression coefficient; Lower, Upper: lower resp. upper 95% confidence limit for regression coefficient. Pr > |t|: p-value; R-Square: 0.19. βHB: oral D-β-hydroxybutyrate ester, SVC: slow vital capacity, NfL: neurofilament light chain, BMI: body mass index, ECM/BCM: extracellular mass/body cell mass, BL: baseline

Effect	Treatment	Estimate	Pr > |t|	Lower	Upper
**Intercept**		-0.00109	0.9852	-0.1183	0.1161
**Group**	Group A: βHB	-0.00391	0.6542	-0.02130	0.01347
**Group**	Group B: Placebo	0	.	.	.
**BL Age**		-0.00049	0.2260	-0.00129	0.000310
**BL SVC**		-0.00033	0.0846	-0.00070	0.000046
**BL NfL**		0.000129	0.0777	-0.00001	0.000272
**BL ECM/BCM**		0.03202	0.1606	-0.01306	0.07711
**BL BMI**		0.000860	0.3378	-0.00092	0.002642

Suppl. Table 12: Results of multiple regression analysis for CNAQ slope models with group, age, baseline NfL, baseline BMI, baseline SVC and baseline CNAQ as covariates.

Estimate: regression coefficient; Lower, Upper: lower resp. upper 95% confidence limit for regression coefficient. Pr > |t|: p-value; R-Square: 0.08. βHB: oral D-β-hydroxybutyrate ester, SVC: slow vital capacity, NfL: neurofilament light chain, BMI: body mass index, CNAQ: Council of Nutrition Appetit Questionnaire, BL: baseline

Effect	Treatment	Estimate	Pr > |t|	Lower	Upper
**Intercept**		2.3722	0.4030	-3.2495	7.9938
**Group**	Group A: βHB	0.5614	0.2166	-0.3363	1.4592
**Group**	Group B: Placebo	0	.	.	.
**BL Age**		-0.01180	0.5774	-0.05383	0.03023
**BL SVC**		-0.00690	0.4536	-0.02516	0.01136
**BL NfL**		-0.00774	0.0521	-0.01555	0.000072
**BL CNAQ**		-0.01751	0.7726	-0.1378	0.1028
**BL BMI**		-0.01131	0.7874	-0.09460	0.07198

Suppl. Table 13: Results of multiple regression analysis for EQ-5D-5L slope models with group, age, baseline NfL, baseline BMI, baseline SVC and baseline EQ-5D-5L as covariates.
Estimate: regression coefficient; Lower, Upper: lower resp. upper 95% confidence limit for regression coefficient. Pr > |t|: p-value; R-Square: 0.32. Bold p-values refer to statistically significant values. βHB: oral D-β-hydroxybutyrate ester, SVC: slow vital capacity, NfL: neurofilament light chain, BMI: body mass index, EQ-5D-5L: life quality questionnaire, BL: baseline

Effect	Treatment	Estimate	Pr > |t|	Lower	Upper
**Intercept**		6.1065	0.1752	-2.7908	15.0038
**Group**	Group A: βHB	1.8611	**0.0399**	0.08888	3.6333
**Group**	Group B: Placebo	0	.	.	.
**BL Age**		-0.04157	0.3261	-0.1255	0.04233
**BL SVC**		0.008324	0.6542	-0.02861	0.04526
**BL NfL**		-0.02479	0.0015	-0.03974	-0.00984
**BL EQ-5D-5L**		-0.07610	0.0004	-0.1172	-0.03498
**BL BMI**		-0.00564	0.9450	-0.1683	0.1570

Suppl. Table 14: Results of multiple regression analysis for free acetone in urine slope model with group, age, baseline NfL, baseline BMI, baseline SVC and baseline free acetone in urine as covariates.
Estimate: regression coefficient; Lower, Upper: lower resp. upper 95% confidence limit for regression coefficient. Pr > |t|: p-value; R-Square: 0.11. βHB: oral D-β-hydroxybutyrate ester, SVC: slow vital capacity, NfL: neurofilament light chain, BMI: body mass index, BL: baseline

Effect	Treatment	Estimate	Pr > |t|	Lower	Upper
**Intercept**		-3.6334	0.8333	-38.0076	30.7408
**Group**	Group A: βHB	2.5337	0.4547	-4.2010	9.2684
**Group**	Group B: Placebo	0	.	.	.
**BL Age**		-0.07614	0.6369	-0.3971	0.2448
**BL SVC**		0.01384	0.8467	-0.1287	0.1564
**BL NfL**		0.05976	0.0512	-0.00031	0.1198
**BL free acetone in urine**		-0.3890	0.2621	-1.0764	0.2983
**BL BMI**		0.1193	0.7096	-0.5186	0.7573

Suppl. Table 15: Results of multiple regression analysis for acetoacetate slope model with group, age, baseline NfL, baseline BMI, baseline SVC and baseline acetoacetate as covariates.
Estimate: regression coefficient; Lower, Upper: lower resp. upper 95% confidence limit for regression coefficient. Pr > |t|: p-value; R-Square: 0.15. βHB: oral D-β-hydroxybutyrate ester, SVC: slow vital capacity, NfL: neurofilament light chain, BMI: body mass index, BL: baseline

Effect	Treatment	Estimate	Pr > |t|	Lower	Upper
**Intercept**		-13.3178	0.6766	-76.8148	50.1791
**Group**	Group A: βHB	6.1491	0.3213	-6.1396	18.4378
**Group**	Group B: Placebo	0	.	.	.
**BL Age**		-0.1905	0.5266	-0.7882	0.4072
**BL SVC**		0.2005	0.1201	-0.05379	0.4548
**BL NfL**		0.1218	0.0272	0.01414	0.2295
**BL Acetoacetate**		-0.08803	0.1335	-0.2037	0.02769
**BL BMI**		0.1723	0.7705	-1.0024	1.3469

Suppl. Table 16: Results of multiple regression analysis for βHB slope model with group, age, baseline NfL, baseline BMI, baseline SVC and baseline βHB as covariates.
Estimate: regression coefficient; Lower, Upper: lower resp. upper 95% confidence limit for regression coefficient. Pr > |t|: p-value; R-Square: 0.15. βHB: oral D-β-hydroxybutyrate ester, SVC: slow vital capacity, NfL: neurofilament light chain, BMI: body mass index, BL: baseline

Effect	Treatment	Estimate	Pr > |t|	Lower	Upper
**Intercept**		-51.8602	0.5700	-233.30	129.58
**Group**	Group A: βHB	20.7528	0.2444	-14.5339	56.0396
**Group**	Group B: Placebo	0	.	.	.
**BL Age**		-0.4997	0.5603	-2.2048	1.2053
**BL SVC**		0.6203	0.0931	-0.1066	1.3471
**BL NfL**		0.3432	0.0293	0.03562	0.6508
**BL βHB**		-0.09863	0.0917	-0.2137	0.01643
**BL BMI**		0.6461	0.6996	-2.6837	3.9759

Suppl. Table 17: Results of multiple regression analysis for total ketone bodies slope model with group, age, baseline NfL, baseline BMI, baseline SVC and baseline total ketones as covariates.
Estimate: regression coefficient; Lower, Upper: lower resp. upper 95% confidence limit for regression coefficient. Pr > |t|: p-value; R-Square: 0.15. βHB: oral D-β-hydroxybutyrate ester, SVC: slow vital capacity, NfL: neurofilament light chain, BMI: body mass index, BL: baseline

Effect	Treatment	Estimate	Pr > |t|	Lower	Upper
**Intercept**		-64.9624	0.5955	-308.18	178.25
**Group**	Group A: βHB	26.7877	0.2614	-20.4430	74.0184
**Group**	Group B: Placebo	0	.	.	.
**BL Age**		-0.6941	0.5464	-2.9806	1.5925
**BL SVC**		0.8210	0.0972	-0.1533	1.7954
**BL NfL**		0.4649	0.0277	0.05257	0.8773
**BL Total ketone bodies**		-0.09562	0.1052	-0.2119	0.02063
**BMI**		0.8228	0.7143	-3.6481	5.2937

<u>**Ulm Nutrition Questionnaire**</u>

1. How many meals do you typically have per day? ☐

2. How many <u>warm</u> meals do you typically have per day? ☐

3. Do you mainly eat vegetarian food? **Yes** ☐ **No** ☐

4. Do you mainly eat vegan food? **Yes** ☐ **No** ☐

5. How often do you eat meat per week? (on average, estimated) ☐☐ times

6. a) How much liquid do you consume per day? ☐.☐ l

   b) What kind of drinks do you consume? (in percent, estimated)

Water	☐☐ %
Coffee	☐☐ %
Juice	☐☐ %
Soft Drinks	☐☐ %
Dairy Drinks	☐☐ %
Alcoholic Drinks	☐☐ %
Other	☐☐ %

7. Do you use any nutritional supplements (e.g. high-caloric drinks)?

   Yes ☐ **No** ☐

- Product ☐
- Volume per day ☐☐☐☐ ml
- Calories per day ☐☐☐☐ kcal

8. What did you eat on your last day <u>at home</u>? (last "typical" meals at home)

Breakfast	
Lunch	
Dinner	
Other / Snacks	

Only for follow-up visits:

9. a) Did you take the study medication in the prescribed daily amount?

   Yes ☐ **No** ☐

b) If no:

☐ discontinued after ☐☐ days

☐ missed doses

Reason for missed doses

10. a) When did you take the study medication?

☐ between meels (as instructed)

☐ with meals

b) How did you take the study medication?

☐ mixed with a drink

☐ pure

11. a) Have your eating habits changed since taking the study medication?

Yes ☐ **No** ☐

b) Have your appetite changed since taking the study medication?

Yes ☐ **No** ☐

c) If yes in a) or b): What has changed?

☐ reduced appetite, but eating habits maintained as instructed

☐ reduced appetite, therefore less food intake than usual

☐ Other:

12. Rate the taste of the study medication on a scale of 1-9
(1=extremely poor taste, 5=indifferent, 9=extremely good taste)
