# Supplementary material for: Efficacy and tolerability of D-β-hydroxybutyrate ester in patients with amyotrophic lateral sclerosis (KETO-ALS)–a randomised, placebo-controlled, double-blind study
Source: eClinicalMedicine. 2026 Oct 26;101:104272. doi: 10.1016/j.eclinm.2026.104272 (PMC13734945; doi:10.1016/j.eclinm.2026.104272)
Supplement: Supplementary file 2 [file mmc2.docx]

**Department of Neurology**

**Study Protocol**

**Efficacy and tolerability of beta hydroxybutyrate ester in patients with Amyotrophic Lateral Sclerosis (ALS)**

**Study center**

Department of Neurology

University of Ulm

Oberer Eselsberg 45

D-89081 Ulm / Germany

phone: +49 (0) 731 177 5285

fax: +49 (0) 731 177 1202

email: johannes.dorst@uni-ulm.de

**Study personnel:**

Prof. Dr. Johannes Dorst, Senior Physician, Department of Neurology (principal investigator)

Prof. Dr. Albert C. Ludolph, Medical Director, Department of Neurology

Christine Herrmann, Assistant Physician, Department of Neurology

Dr. Simon Witzel, Assistant Physician, Department of Neurology

Dr. Joachim Schuster, Head of Clinical Study Center, Department of Neurology

Andrea Weber, Student of Medicine

Natalie Schön, Study Nurse

Sabine Raubold, Study Nurse

Christina Hecht, Study Nurse

Sarah Baumeister, Study Nurse

Elke Fröhlich, Study Nurse

**Biometry**

Institute for Epidemiology und Medical Biometry

University of Ulm

Dr. Jens Dreyhaupt

Schwabstr. 13

89075 Ulm

phone: +49 (0) 731 50 26895

fax: +49 (0) 731 500 26902

email: jens.dreyhaupt@uni-ulm.de

**1.	SYNOPSIS**

| **TITLE OF STUDY** | Efficacy and tolerability of beta hydroxybutyrate ester in patients with Amyotrophic Lateral Sclerosis (ALS) |
| --- | --- |
| **SHORT TITLE** | KETO-ALS2 |
| **PRINCIPAL INVESTIGATOR** | Prof. Dr. Johannes Dorst  University of Ulm, Department of Neurology  Oberer Eselsberg 45, D-89081 Ulm, Germany  +49 731 177 5285 / johannes.dorst@uni-ulm.de |
| **STATISTICIAN** | Dr. Jens Dreyhaupt  University of Ulm, Institute of Epidemiology and Medical Biometry  Schwabstr. 13, D-89075 Ulm, Germany  +49 731 50 26895/ jens.dreyhaupt@uni-ulm.de |
| **CONDITION** | Amyotrophic Lateral Sclerosis (ALS) |
| **HYPOTHESIS** | Beta hydroxybutyrate ester lowers neurofilament light chain (NfL) serum levels as a negative prognostic biomarker in ALS |
| **STUDY POPULATION** | Key inclusion criteria:  - Probable (clinically or laboratory) or definite ALS according to the revised version  of the El Escorial World Federation of Neurology criteria^1^  - Loss of ALS functional rating scale revised (ALSFRS-R) of ≥ 0.33 points per month since onset (first paresis) based  on the formula:  (48 – Score at Screening Visit) / (Months between Onset and Screening Visit)  - Age ≥18 years  - Continuously treated with 100 mg riluzole per day for at least 4 weeks  - Capable of thoroughly understanding all information given and giving full  informed consent according to GCP  Key exclusion criteria:  - Hyperinsulinism  - Pyruvate decarboxylase deficit  - Disturbance of fatty acid oxidation  - Disturbance of gluconeogenesis  - Acute porphyria  - Metabolism disorders which prevent utilization or degradation of ketone bodies  - Severe gastro-esophageal reflux  - Renal insufficiency (medical history and/or elevated serum creatinine levels and/or GFR <90 ml/min)  - Previous participation in another interventional study within the preceding 4 weeks  - Tracheostomy  - Pregnancy or breast-feeding females  - Evidence of a major psychiatric disorder or clinically evident dementia  - Intake of diuretics  - Severe dysphagia  - Nutrition via percutaneous endoscopic gastrostomy (PEG)  - Electrolyte or acid-base imbalance  - Heart Failure NYHA II or above |
| **STUDY TYPE** | 1:1 randomized, prospective, stratified, parallel-group, double-blind,  placebo-controlled study |
| **TREATMENTS** | Group A: beta hydroxybutyrate ester, 3x20 ml KE4® (KetoneAid, Falls Church,  USA) per day, corresponding to 3x 10 g beta hydroxybutyrate ester;  n=38, oral application  Group B: placebo, n=38, manufactured by Ulm University Pharmacy,  oral application    Both treatments will be administered in addition to standard of care (riluzole 100 mg/day and high-caloric nutrition). |
| **Endpoints** | Primary endpoint:  Change of neurofilament light chains (NfL) in serum after 6 months compared to baseline. The change will be measured as individual NfL slope from baseline to 6 months (change per month).  Secondary endpoints:  - Survival (time to death or tracheostomy) after 6 months  - ALS functional rating scale revised (ALSFRS-R)  - ALS functional rating scale revised (ALSFRS-R) self-rating via app  - Body mass index (BMI)  - Slow vital capacity (sVC)  - Resting energy expenditure (REE)  - body composition (bioelectrical impedance analysis; BIA)  - Change of individual quality of life (EQ-5D-5L)  - beta hydroxybutyrate in serum  - acetone in urine  - Council of nutrition appetite questionnaire (CNAQ)  - Ulm nutrition questionnaire (UNQ)  - Terms and frequencies of adverse eves (AEs) and serious adverse events  (SAEs)  All secondary endpoints (except survival) will be measured as changes to baseline after 6 months. |
| **TRIAL DURATION** | First patient in to last patient out: 18 months  Duration of the entire study: 24 months  Recruitment period: 12 months |
| **STATISTICAL**  **ANALYSIS** | Statistical methods used to compare groups for primary and secondary outcomes:  Efficacy: Two-sample t-test or Wilcoxon rank sum test as appropriate, one-sided significance level of 5%  Description of the primary efficacy and population: To analyze NfL serum levels under therapy, we will calculate the slopes from a univariate linear regression model separately for each patient. Group comparisons of NfL slopes will be done using the two-sample t-test or Wilcoxon rank sum test as appropriate. Additionally, a one-sided 95% confidence interval will be provided as effect estimation for the difference of group means or group medians of NfL slopes. Furthermore, multiple linear regression models will be applied to adjust for potential confounders.  Safety: Adverse Events will be coded by MedDRA terminology and analyzed by evaluating absolute and relative frequencies.  Secondary endpoints: Descriptive statistics will be provided. For continuous outcomes, the two-sample t-test or the Wilcoxon rank sum test will be used for analysis of group differences as appropriate. For group differences of survival, Kaplan Meier curves and log-rank test will be used. For ALSFRS-R, the slopes (loss of points per month) during treatment will be analyzed as well as the deviation of slopes from an NfL prediction model (Witzel et al., submitted for publication).  Subgroup analyses: Patients with fast and very fast disease progression, i.e., patients with an ALSFRS-R slope per month < / ≥ median and < / ≥ upper third (both pre-baseline and during the study), patients with NfL baseline values < / ≥ median and < / ≥ upper third; patients with NfL slopes < / ≥ median and < / ≥ upper third during the study ; patients with and without loss of BMI (both pre-baseline and during the study)  Exploratory analysis of primary endpoint, all analyses of secondary endpoints, and safety analysis are explorative at a significance level of 0.05 (two-sided). |
| **SAMPLE SIZE** | To be assessed for eligibility (n = 84)  To be allocated to trial (n = 76)  To be analysed (n = 76) |

**2.	RATIONALE FOR THE TRIAL DESIGN**

**2.1	Scientific background**

Amyotrophic lateral sclerosis (ALS) is a severe neurodegenerative disease characterized by progressive degeneration of the anterior horn cells of the spinal cord, their homologues in the brain stem and the upper motor neurons in the primary motor cortex. Clinically, the disease progresses from initial asymmetric weakness, wasting and atrophy to complete loss of spinal and bulbar motor functions. Mean survival of ALS patients is about 2–5 years. Death is most frequently related to progressive worsening of respiration due to affection of respiratory muscles. The ALS Research Committee of the World Federation of Neurology has established research diagnostic guidelines, the El Escorial criteria^1^^,^^2^. ALS is considered an orphan disease; its incidence is estimated with 1.5 –3/100000/year and prevalence rates are 3-6/100,000^3^. Treatment of ALS remains unsatisfactory; riluzole, a benzothiazole derivative and glutamate release blocker, has been approved for use in ALS because of its modest effect on survival^4^. The drug is generally well tolerated, although liver toxicity must be controlled. Riluzole is the only approved drug for treatment of ALS in Europe.

Weight loss is a common phenomenon and an independent negative prognostic factor in ALS^5^^,^^6^. Several potential pathogenetic mechanisms, including intrinsic hypermetabolism^7^ and deficient food intake^8^, have been discussed in this context. Basic research on animal models provided compelling evidence that nutritional cues are able to modulate the course of ALS^9^ . Mice models of ALS show prominent leanness^9^ caused by increased energy expenditure and muscle hypermetabolism. Conversely, increasing lipid intake in ALS animal models compensates their energy deficit, increases their lifespan and delays motor neuron degeneration, while caloric restriction hastens disease endpoint^9^. Thus, in animal models, available evidence suggests that energy deficit, as a consequence of ALS pathogenesis, is actively participating in the progression of motor neuron degeneration^10^.

These pathogenic features of ALS models are strongly reminiscent of available clinical data in humans. Indeed, several typical features of ALS patients indicate caloric imbalance. First, ALS patients are usually lean, and their BMI is normal or low^11^. Second, weight loss and loss of body fat is a typical feature of patients during disease progression^5^. This progressive loss of energy stores is due to several mechanisms, including dysphagia for patients experiencing bulbar symptoms, and increased energy expenditure as repeatedly observed using indirect calorimetry. The pathogenic background of hypermetabolism in ALS is largely unknown; however, various studies have demonstrated a dysfunction of mitochondrial complex I^12^ which is essential in order to establish the electrochemical potential difference across the inner mitochondrial membrane needed for production of adenosine triphosphate (ATP). Importantly, a large body of evidence unequivocally demonstrates that malnutrition, BMI and weight loss are among the most prominent negative prognostic factors in ALS^5^^,^^6^^,^^10^^,^^13^.

Consistently, recent studies aiming at targeting weight loss and cachexia in ALS have shown promising results. In a pilot study conducted in 2013, we demonstrated that body weight can be stabilized in ALS by applying either a fat-rich or carbohydrate-rich high-caloric food supplement^14^. One year later, a small placebo-controlled randomized controlled trial (RCT) showed that a carbohydrate-rich, hypercaloric diet extended survival in patients fed via percutaneous endoscopic gastrostomy (PEG)^15^, while we were able to show that high-caloric nutrition (i.e., daily intake of ≥1500 kcal) had a life-prolonging effect in a German nation-wide registry study of PEG patients^16^. Last year, we concluded a placebo-controlled multicenter RCT (LIPCAL-ALS^17^^,^^18^) in 201 patients within the German network for motor neuron diseases (MND-NET) in order to evaluate a potential life-prolonging effect of a high-caloric, high-fat food supplement (+405 kcal/day, 100% fat in addition to normal food intake) which revealed a strong survival benefit for fast-progressing patients. Fast-progressing patients are defined by the loss of amyotrophic lateral sclerosis functional rating scale revised (ALSFRS-R^19^; the standard clinical score measuring functional deficits and disability of patients with ALS^19^) over time upfront baseline/randomization. In LIPCAL-ALS, patients who had a loss of ALSFRS-R above the median before baseline (>0.62 points/month), showed a survival probability of 0.62 (95% CI 0.47–0.74) after 18 months in the intervention group, while placebo patients showed a survival probability of 0.38 (95% CI 0.21–0.54), which was statistically significant (HR 0.50, two-sided 95% CI 0.27–0.92, p=0.02)^20^. In the same subgroup, loss of body weight was less extensive in the intervention group (-0.10 points of BMI per month, IQR 0.04– -0.25) compared to placebo (-0.28 points of BMI per month, IQR -0.01– -0.44; p=0.02)^20^. Furthermore, in the whole study population, disease progression as measured by loss of ALSFRS-R score per month was gradually and significantly slowed down in the intervention group during the study (p<0.01) as opposed to the placebo group which displayed a stable disease progression^18^. These positive clinical findings in the intervention group were accompanied by a significant decline of neurofilament light chains (NfL) serum levels^18^ as the standard prognostic biomarker in ALS^21^^,^^22^, signifying the extent of axonal damage during a defined period of time.

**2.2	Trial rationale**

ALS is seen as a rare disease; however, current incidence rates in the industrialized world of 2.5-3/100,000 imply that every 500th German citizen will die from ALS. Therefore, effective therapies that will slow down or stop this devastating disease are desperately needed.

The LIPCAL-ALS results^17^^,^^18^ suggest that ALS patients, especially those with a fast-progressing course of disease, might experience an extensive benefit with regard to stabilization of body weight, disease progression, and survival, by therapeutically targeting the energy deficit. However, to date it remains unknown which exact high-caloric food composition yields the largest benefit. Based on the pathophysiological background explained above, we hypothesize that the most effective substrate is a compound which offers the maximum amount of energy while at the same time providing an acceptable tolerability profile in order to assure sufficient compliance and quality of life.

Based on these considerations, ketone bodies represent a promising therapeutic option as they constitute an extremely high-energetic substrate which can cross the blood brain barrier via a specific, physiological transporter. Under standard conditions, the central nervous system (CNS) utilizes glucose as the main substrate in order to produce energy in the form of ATP. However, under fasting conditions, the liver synthesizes ketone bodies (β-hydroxybutyrate (βHB), acetoacetate, and acetone) from fat which provide a significantly higher amount of ATP per mole compared to glucose (figure 1).

***Figure 1****: In the liver, fatty acids are ordinarily converted into acetyl-CoA which enters the tricarboxylic acid (TCA) cycle. When fatty acid levels are elevated and exceed the metabolic capacity of the TCA cycle, acetyl-CoA is shunted to ketogenesis. Two acetyl-CoAs can combine through a thiolase enzyme to produce acetoacetyl-CoA, which is a precursor for the synthesis of acetoacetate (ACA) and β-hydroxybutyrate (BHB). Acetone, the other major ketone body, is produced primarily from spontaneous decarboxylation of ACA, and can be eliminated as a volatile substrate through the lungs and kidneys. In the blood, ACA and BHB are transported from the vascular lumen to the brain interstitial space, and to both glia and neurons, by monocarboxylic acid transporters (MCTs). MCT-1 is the principal carrier localized to the vascular endothelium. Within neurons, both ACA and BHB are transported directly into mitochondria, and then converted to acetyl-CoA through several enzymatic steps. BHB is converted to ACA through D-β-hydroxybutyrate dehydrogenase, and ACA undergoes subsequent conversion to acetoacetyl-CoA through a succinyl-CoA transferase enzyme. Finally, acetoacetyl-CoA-thiolase converts acetoacetyl-CoA to two acetyl-CoA moieties which then enter the TCA cycle. Abbreviations: CAT (carnitine-acylcarnitine translocase), FAO (fatty acid oxidation), ACA (acetoacetate), BHB (β-hydroxybutyrate), MCT-1 (monocarboxylate transporter-1), GLUT-1 (glucose transporter-1), BBB (blood-brain barrier), CPT-1 (carnitine palmitoyl transferase), UCP (uncoupling protein), ATP (adenosine triphosphate), ❶(3-hydroxybutyrate dehydrogenase), ❷ (succinyl-CoA3-oxoacid CoA transferase), ❸ (mitochondrial acetoacetyl-CoA thiolase). MRC, mitochondrial respiratory complex; figure taken from Kim et al., Curr Opin Clin Nutr Metabl Care 2008**^23^*

This metabolic transition is the basic principle of the ketogenic diet, a carbohydrate-free, fat-rich diet, which has shown beneficial effects in other neurodegenerative diseases such as Alzheimer’s disease^24^ and Parkinson’s disease^25^ and was also effective in a mouse model of ALS^26^. In this model, mice treated with a ketogenic diet showed a significantly slower decline of motor function (figure 1), i.e., the time until loss of 50% of motor function was significantly delayed in mice fed with ketogenic diet (from 85 to 136 days of age). Of note, animals fed with a ketogenic diet also gained body weight compared to controls (Figure 2). Importantly, the results of this study do not necessarily imply that the direct application of ketone bodies has the same effects, since – as opposed to a ketogenic diet – glucose is still available for the body. However, since the main rationale for this study is based on the availability of an alternative, high-energetic substrate, it is reasonable to assume similar clinical effects. Although it may be suspected that, similar to glucose, the utilization of ketone bodies might also be impaired in ALS, this animal study suggests that the relative gain of energy per mole compared to glucose may induce beneficial clinical effects.

***Figure 2****: In the transgenic ALS mouse model, mice fed with ketogenic diet compared to controls showed increased ketone body serum levels (A), higher serum concentrations of beta hydroxybutyrate (B), a slower loss of motor function (C; time to failure = time until loss of 50% of baseline motor function), and higher body weight (D); p<0.05; figure taken from Zhao et al., BMC Neurosci 2006**^26^*

A ketogenic diet is extremely difficult to establish in ALS patients as it requires a long-term change of eating habits which is hindered by dysphagia, the rapidly declining general condition and the massively reduced life expectancy. However, the direct application of ketone bodies may constitute an effective and easy-to-apply alternative compared to a ketogenic diet. In recent years, it has been convincingly shown that by applying ketone bodies directly, either via salts or esters, significantly increased ketone body serum levels can be achieved and sustained for several hours in humans^27^^,^^28^. It has been shown repeatedly that the intake of ketone bodies, even in high dosages, is completely safe and showed acceptable tolerability^28-30^, although minor gastrointestinal symptoms have been frequently reported, which in this study will be addressed by various measures described below.

Therefore, we hypothesize that the energy deficit in ALS as an important negative prognostic factor can be targeted by administering ketone bodies as a high-energetic substrate, and that this intervention may slow down disease progression and lower NfL serum levels. Importantly, ketone bodies (or placebo) will be administered in addition to standard therapy in ALS including high-caloric nutrition. By maintaining sufficient caloric intake and at the same time offering an alternative, more efficient substrate for generating energy, we hypothesize that the high-energetic add-on can beneficially utilized in damaged neurons by sustaining reparation processes and trigger anabolic processes which counteract cachexia and weight loss in ALS.

**2.3	Treatments and rationale for dose selection**

Patients eligible for study participation will be randomized to one of two groups (ketone bodies or placebo) at baseline visit. Ketone bodies or placebo will be administered as an add-on to the standard therapy with riluzole and high-caloric nutrition. Standardized questionnaires (see 3.4) will be applied to assure that the intervention does not interfere with the recommended high-caloric nutrition and that recommended eating habits are maintained throughout the study.

Intervention group:

Patients in the intervention group will receive 20 ml (10 g) of β-HB ester (*KE4®, KetoneAid*, Falls Church, USA) 3 times daily. Patients will be instructed to take the study medication in the morning, in the afternoon and in the evening with maximum distance (at least 1 hour) to meals (e.g.: meals at 8 am, 12 pm, and 6 pm, intervention at 10 am, 3 pm, and 9 pm). This is important since food intake (especially carbohydrates) may lower ketone body serum levels^28^. For the exact composition of the product, see table 1. Patients will be instructed to otherwise maintain their usual eating habits, which includes a high-caloric nutrition as generally recommended for ALS. This will be controlled by a standardized questionnaire (Ulm nutrition questionnaire^17^).

| **Content per dosage of KE4 (20 ml)** |  |
| --- | --- |
| Ingredients | D-Beta-Hydroxybutyrate Ketone Ester  Water  Citric Acid  Allulose  Organic Stevia  Natural Flavors  Potassium Sorbate |
| Calories | 48 |
| Fat | 0% |
| Cholesterol | 0% |
| Sodium | 12 mg |
| Carbohydrates | 167 mg |
| Protein | 0% |
| Potassium | 33 mg |
| pH | 4.6 |

***Table 1****: Content of Study Intervention.*

Placebo group:

Patients in the placebo group will receive 20 ml of a matching placebo solution (similar look and taste) 3 times daily. Placebo will be manufactured by the pharmacy of Ulm University. Similar to the intervention group, patients will be instructed to otherwise maintain their usual eating habits, which implies a high-caloric nutrition as generally recommended for ALS. This will be controlled by a standardized questionnaire (Ulm nutrition questionnaire^17^).

| **Placebo Composition** |  |
| --- | --- |
| Ingredients | Glucose Solution 50%  Bitter Orange Peel Tincture (3 drops/ 200 ml)  Ginger Tincture (3 drops/ 200 ml) |
| Calories | 40 |

Rationale for dose selection:

The intervention in this study (*KE4®, KetoneAid*, Falls Church, USA) is commercially available and predominantly used by athletes who aim at improving their performance by increasing available energy levels. For this purpose, β-HB ester is usually consumed in a dosage of 1-4 x 10-15 ml per day which is the recommended maximum dosage as per label. While preparing this study, we tested various dosages of the product in 6 healthy probands (self-experiment) and found a slightly higher dosage of 3 x 20 ml to be most effective in order to maintain high β-HB serum levels while at the same time providing acceptable tolerability. Subsequently, we conducted an individualized treatment with this dosage in

2 patients over 2 days and found that β-HB serum levels increased significantly up to maximum values of 3.7 mg/dl about 10 minutes after intake, corresponding to about the 8-fold baseline concentration. Over the following time, β-HB serum levels decreased again and reached baseline levels 1–2 hours before the next intake (figure 3).

***Figure 3****: Example of β-HB serum levels measured in an ALS patient during intake of a daily dosage of 3x20 ml beta hydroxybutyrate ester on day 1 (blue) and day 2 (red). The other patient showed similar curves. Black arrows indicate the intake of beta hydroxybutyrate ester; brown arrows indicate meals.*

Overall, the tolerability of treatment was very good. There were no side effects. The results of this testing phase are therefore in line with a previous study using the same product^29^ which reported mild side effects (mostly gastrointestinal) in 4/12 healthy subjects taking similar dosages over 5 days.

**2.4. Measures to minimize risks and improve tolerability**

The following measures have been implemented to minimize risks and improve tolerability: At baseline, patients will be extensively informed about potential tolerability problems (e.g., diarrhea, change of stool consistency, mild abdominal pain) and about measures to improve tolerability such as mixing the intervention with water. After 3 days and 2 weeks, we will conduct phone visits in order to carefully evaluate tolerability issues. The above mentioned measures to improve tolerability will be controlled during the phone visits. In case of persisting tolerability problems which either constitute a clinically relevant risk or a burden for the patient, the intervention will be stopped. A termination of the intervention will be considered after 2 weeks (second phone visit). Additionally, an unscheduled on-site visit will be offered including a physical examination, safety blood analysis (blood count, electrolytes, liver and kidney values, CRP), and additional diagnostics as necessary. An interim safety analysis will be performed when 50% of patients have completed V2 (=half of the study duration after 3 months), and termination criteria for the study have been defined (see 3.6).

**2.5	Summarized risk-benefit assessment**

ALS is a devastating disease which dramatically reduces life expectancy and physical well-being. The potential risks of every treatment of ALS must be seen against this background.

*KE4®* is a ketone body supplement which is marketed internationally and is freely available. To date, no relevant adverse events have been described. The product has been previously used in a clinical study^28^ which found short-term effects on various laboratory values (increase of insulin, decrease of glucose, potassium, and pH) using an about 2-fold dosage compared to this study. These alterations were (with the exception of a temporary hypokalemia and hyperchloridemia) within the physiological normal ranges. Given a normal renal function, medically relevant alterations of electrolyte serum levels are not expected. Another study with the same product in a similar as well as two higher dosages found only mild side effects, mostly gastrointestinal problems during 5 days of treatment. In the group receiving a similar dosage compared to this study, these mild side effects affected 4/12 subjects.

Furthermore, KE4 has also been used in our clinic during an individualized treatment in 2 patients with ALS over 2 days (see above). During this treatment, we did not record any significant laboratory changes in any patient as controlled by daily laboratory testing, including blood count, electrolytes, liver and kidney values, and inflammation markers. Therefore, we consider the intervention to be safe with an appropriate risk to benefit ratio.

However, to date, KE4 has not been tested over a prolonged period of time as intended in this trial. Therefore, patients will be extensively informed about the potential occurrence of previously unknown long-term side effects.

**3.	TRIAL DESIGN**

**3.1	Trial duration and schedule**

The estimated duration of the whole study is 24 months: 12 months for recruitment, 6 months for treatment and follow-up visits, and 6 months for data cleaning, evaluation, and preparation of the final study report. The recruitment shall start in Q3 2021, therefore the anticipated end of the study is Q3 2023. The actual study duration and recruitment period may deviate from this estimation.

**3.2	Number of patients**

N=76 patients will be recruited, 38 per treatment arm. This number covers a drop-out rate of 5.5% and a death rate of 5.5% as seen in LIPCAL-ALS^17^. Furthermore, patients who drop out during the first 4 weeks of the study (e.g., due to intolerance of the study intervention) will be compensated by additional recruitment. However, all patients randomized will be analyzed according to the intention-to-treat principle. In consideration of an estimated share of 10% unsuccessful screenings, n=84 patients have to be screened.

**3.3	Primary outcome**

Primary endpoint is the change of NfL serum levels after 6 months compared to baseline. The changes will be measured as individual NfL slope from baseline to 6 months (loss of NfL per month), calculated for each patient using an univariate linear regression model.

**3.4	Secondary outcomes**

- survival (time to death or tracheostomy) after 6 months

- ALS Functional Rating Scale Revised (ALSFRS-R): a validated 12-item rating instrument for monitoring the

progression of disability in patients with ALS^19^.

- ALS Functional Rating Scale Revised (ALSFRS-R) self-rating via app (Ambulanzpartner Soziotechnologie APST

GmbH, Berlin, Germany; optional)

- Body mass index (BMI)

- Slow vital capacity (sVC)

- Resting energy expenditure (REE) measured by indirect calorimetry; a non-invasive procedure which allows to

calculate heat production by measuring carbon dioxide and oxygen in the expired air using a canopy hood

- Body composition measured by bioelectrical impedance analysis (BIA); a non-invasive procedure used to analyze

body composition, including fat mass, total body water, muscle mass, fat free mass, body cell mass, extracellular

mass, and lean body mass

- Change of individual quality of life (EQ-5D-5L)

- Beta hydroxybutyrate in serum

- Acetone in urine

- Council of nutrition appetite questionnaire (CNAQ): a validated 8-item tool to evaluate appetite^31^

- Ulm Nutrition Questionnaire (UNQ): a standardized questionnaire to monitor changes of eating habits which was

validated in the LIPCAL-ALS study^17^

- Terms and frequencies of adverse events (AEs) and serious adverse events (SAEs)

All secondary endpoints except survival will be measured as changes after 6 months compared to baseline.

**3.5	Measures taken to minimize / avoid bias, Randomization, Stratification**

The study will take place at the Department of Neurology, University Clinic Ulm, which is specialized in diagnosis and treatment of ALS patients. Inclusion and exclusion criteria represent the ALS population while at the same time facilitating smooth enrollment. After excluding contraindications against administration of ketone bodies and informed consent, patients are randomly assigned to one of the treatment arms. The randomization list will be generated by the Institute of Epidemiology and Medical Biometry Ulm University, Germany, using a validated system, which involves a pseudo-random number generator to ensure that the resulting treatment sequence will be both reproducible and non-predictable^32^. Through this, medical safety as well as minimization of bias can be assured. Central randomization will be administered in a blinded fashion. Blinding will be done by the pharmacy of the University Hospital Ulm. In order to ensure an equal group composition with regard to relevant prognostic factors, a stratification as per status of weight loss prior to baseline (yes/no within the preceding 6 months) and progression rate (cutoff loss of ALSFRS-R ≤0.77 versus >0.77 points per month at baseline; 0.77 was the median in LIPCAL-ALS^17^ for the subgroup of patients with a pre-baseline ALSFRS-R- slope of ≥0.33 points/month) will be carried out. A placebo arm will ensure equal treatment conditions for both groups and control for placebo effects. Additionally, it is intended to prevent patients from purchasing the intervention themselves, which is commercially available (as a potential risk associated with an open control group).

Trial endpoints are validated disease addressing scales and measurements which will be collected in a controlled and standardized form. Raters will be ALS specialists with training and experience in the conduct of clinical trials. All raters will be trained before the start of the study. Patients will be examined by the same raters during the whole study. Raters, treating physicians and patients will be blinded. Standardized dietary questionnaires (CNAQ, UNQ) will ensure that results will not be influenced by changing eating habits caused by the intervention and enable to monitor compliance.

**3.6	Selection and withdrawal of patients**

Each patient can only once be included into the study.

Inclusion criteria:

1. Probable (clinically or laboratory) or definite ALS according to the revised version of the El Escorial World

Federation of Neurology criteria^1^

2. Loss of ALS functional rating scale revised (ALSFRS-R) of ≥ 0.33 points per month since onset (first paresis) based on the formula:

(48 – score at screening visit) / (months between onset and screening visit)

3. Age ≥18 years

4. Continuously treated with 100 mg riluzole per day for at least 4 weeks

5. Capable of thoroughly understanding all information given and giving full informed consent according to GCP

The progression rate criterion (2) will assure that patients will atypical slow progression (e.g. subforms / special variants like primary lateral sclerosis) will not enter the study. As shown in LIPCAL-ALS^17^, patients with slow progressive forms are not responding to high-energetic nutritional interventions and/or their disease progression is too slow in order to display a clinically relevant effect within the timeframe of the intervention. The cut-off of a loss of

≥ 0.33 ALSFRS-R points per month excludes about 25% of patients according to LIPCAL-ALS^17^.

Exclusion criteria:

1. Hyperinsulinism

2. Pyruvate decarboxylase deficit

3. Disturbance of fatty acid oxidation

4. Disturbance of gluconeogenesis

5. Acute porphyria

6. Metabolism disorders which prevent utilization or degradation of ketone bodies

7. Severe gastro-esophageal reflux

8. Renal insufficiency (medical history and/or elevated serum creatinine levels and/or GFR <90 ml/min)

9. Previous participation in another clinical study within the preceding 4 weeks

10. Tracheostomy

11. Pregnancy or breast-feeding females

12. Evidence of a major psychiatric disorder or clinically evident dementia precluding evaluation of symptoms.

Disorders listed under 1–7 represent known contraindications against a ketogenic diet and might therefore also cause complications when administering ketone bodies. Patients with severe renal insufficiency (8) will be excluded because of the distorting effect on NfL values.

13. Intake of diuretics

15. Nutrition via percutaneous endoscopic gastrostomy (PEG)

16. Electrolyte or acid-base imbalance

17. Heart Failure NYHA II or above

Patients may withdraw their consent at their own request without given reasons at all times during the study without any disadvantages regarding their regular treatment. Reasons for withdrawal will be recorded if the patient agrees. Whenever possible, all patients prematurely discontinuing study participation should perform a termination visit including the scheduled evaluations. If the follow-up visits cannot be performed on the intended dates, a visit should be performed on an earlier or later time whenever possible, and the reasons for the adjournment should be documented as far as they are known.

A subject must be withdrawn from study treatment for any of the following reasons:

- The subject experiences a medical emergency that necessitates permanent discontinuation of study treatment.

- The subject experiences ongoing tolerability problems (e.g. diarrhea) which either constitute a clinically relevant risk according to the treating physician and/or a significant burden for the patient.

- The subject withdraws consent to continue study treatment.

- The subject becomes pregnant.

- At the discretion of the Investigator.

The reason for discontinuation of study treatment must be recorded in the study files.

The whole study may be discontinued if new risks for patients become known for the treatment, if a disadvantage of the treatment becomes apparent or if serious adverse events occur which are caused by the intervention. After 50% of patients have completed V2 (=50% of intervention time), an interim safety analysis will be conducted by an independent unblinded statistician, including adverse events, survival status, neurofilament serum levels, body weight, and ALSFRS-R. The study will be terminated if any disadvantages for patients receiving beta hydroxybutyrate (e.g. a higher loss of body weight since baseline compared to placebo) becomes apparent or if the analysis yields an unfavorable risk to benefit ratio. Furthermore, the study will be terminated at any time if persisting and clinically relevant tolerability issues (as evaluated by the principal investigator) become apparent in a significant share of patients. During interim safety analysis, recruitment will be continued. Finally, difficulties in the recruitment of patients may cause a premature closure of the study. In any case, the ethics committee will be informed.

**3.7	Trial schedule**

| **Visit** | **V1**  **baseline** | **T1**  **3 days** | **T2**  **2 weeks** | **V2**  **3 months** | **V3**  **6 months** |
| --- | --- | --- | --- | --- | --- |
| Informed consent | x |  |  |  |  |
| Medical history / Medication | x |  |  | x | x |
| Adverse events |  | x | x | x | x |
| Physical and neurological examination | x |  |  | x | x |
| ALSFRS-R | x | x | x | x | x |
| ALSFRS-R self-rating via App (optional) | x | x | x | x | x |
| BMI | x | x | x | x | x |
| sVC | x |  |  | x | x |
| REE | x |  |  | x | x |
| BIA | x |  |  | x | x |
| EQ-5D-5L* | x |  |  | x | x |
| CNAQ | x | x | x | x | x |
| Ulm Nutrition Questionnaire | x | x | x | x | x |
| Blood sample (safety) | x |  |  | x | x |
| Blood sample (scientific; optional) | x |  |  | x | x |
| NfL (serum) | x |  |  | x | x |
| βHB (serum) | x |  |  | x | x |
| Acetone (urine) | x |  |  | x | x |

Baseline visit (V1): After verification of inclusion and exclusion criteria, the patient will be informed about the study and an informed consent will be obtained. The medical history and concomitant medication will be obtained and a physical and neurological examination will be conducted. Clinical scores (ALSFRS-R, EQ-5D-5L, CNAQ, UNQ) will be obtained. BMI, sVC, REE, and body composition will be measured. Venous blood sampling (blood count, coagulation, electrolytes, CRP, liver and kidney values, NfL, βHB) and urine analysis (acetone) will be done. The patient may agree that two additional serum monovettes are taken for scientific purposes and may sign a transfer agreement form. The patient may also agree to participate in an optional ALSFRS-R self-rating via app.

Patients will be randomized to receive either the intervention or placebo and receive the respective study medication.

Telephone visits (T1–T2): We will perform a phone call after 3 days and 2 weeks to assure adequate tolerability and compliance. In case of tolerability problems, the patient will again be instructed how to minimize gastrointestinal problems. In case of persisting significant side effects which constitute a medical risk as evaluated by the treating physician and/or a burden for the patient, the intervention will be terminated. In case of side effects, an unscheduled on-site visit including physical examination, blood analysis (blood count, electrolytes, liver and kidney values, CRP), and additional diagnostic measures as need, will be offered.

Follow-up visits (V2–V3): Medical history, concomitant medication, physical and neurological examination, ALSFRS-R, ALFRS-R (self-rating), BMI, sVC, REE, EQ-5D-5L, CNAQ, UNQ, venous blood sampling (blood count, coagulation, electrolytes, CRP, liver and kidney values, NfL, βHB), and urine analysis (acetone) will be done at both follow-up visits. Additionally, adverse events will be recorded at every visit. After V2, an interim safety analysis will be performed (see above).

**4.	DATA MANAGEMENT, QUALITY MANAGEMENT AND ETHICAL ASPECTS**

Data Management: Pseudonymized study data will be documented in the patient records (physician letter) and study data base. The study data base is secured by password and can only be accessed by authorized staff based on a list which is generated by the principle investigators before the beginning of the study. All data are subject to the local data protection regulations. The treating physician is responsible for the correct data entry and for the correction of incorrect data. The principle investigators and persons who are authorized by the principle investigators will check the data for plausibility.

Patient information and informed consent: Before being admitted to the clinical study, the patient must consent to participate after being fully informed about the nature, scope, and possible consequences of the study. The documents are in a language understandable to the patient and specify who informed the patient. After reading the informed consent document, the patient must give consent in writing. The patient's consent must be confirmed by the personally dated signature of the patient and by the personally dated signature of the study physician conducting the informed consent discussions. A copy of the signed informed consent document must be given to the patient. The original signed consent document will be retained by the investigator. Without signed informed consent, no study-related procedure will be applied. The patient’s family doctor will be informed if the patient does agree. If the patient is unable to personally date and sign the consent form due to physical disability, consent must be confirmed orally in the presence of an impartial witness. The witness and the person conducting the informed consent discussions must sign and personally date the consent document.

Responsibilities of principal investigators: The principal investigator ensures that all persons assisting with the study are adequately informed about the protocol, the study procedures, and their study-related duties and functions.

Confidentiality: The name of the patients and other confidential information are subject to medical professional secrecy. Pseudonymized study data may only be transferred to other persons after the treating physicians have been released from professional secrecy by the patient.

Ethical and legal aspects: The procedures set out in this study protocol, pertaining to the conduct, evaluation, and documentation of this study, are designed to ensure that all persons involved in the study abide by good clinical practice (GCP) so far applicable and the ethical principles described in the Declaration of Helsinki.

Approval of study protocol: Before the start of the study, the study protocol and the informed consent documents are submitted to the responsible Ethics Committee. Before the first patient is enrolled in the study, all ethical and legal requirements must be met. The Ethics Committee will be informed of all subsequent protocol amendments which might require formal approval.

Report: After conclusion of the study, a clinical study report shall be written by the principle investigator. The report will include a statistical analysis and an appraisal of the results from a medical viewpoint. It will be based on the items listed in this study protocol.

Publication: All rights to publish the results in articles, newspapers, medical journals, and oral presentations are reserved by the Department of Neurology, University Hospital Ulm. It is planned to publish the results of the study as an original article in an appropriate medical journal as well as presentations at congresses.

Funding: This study is an investigator-initiated trial of the University of Ulm, with financial support from a private donation. The donor is privately connected to ALS and intends to support ALS research. He has outlined his intentions in a letter submitted to the compliance office of the University Clinic Ulm which accepted the donation. He has no relationship to *KetoneAid (Falls Church, USA)*, the manufacturer of the intervention.

**5.	STATISTICAL ANALYSIS AND POWER CALCULATION**

**5.1 Statistical analysis**

Efficacy: To analyze NfL serum levels under therapy, we will calculate the individual slopes from a univariate linear regression model separately for each patient. Group comparisons of NfL slopes will be done using the two-sample t-test or the Wilcoxon rank sum test as appropriate at a one-sided significance level of α = 0.05. Additionally, a one-sided 95% confidence interval will be provided as effect estimation for the difference of group means or group medians. Furthermore, multiple linear regression models will be applied to adjust for potential confounders. Replacement of missing values in the primary outcome (NfL slope) is not possible and will not be done. Analyses will be based on all randomized patients (intention-to-treat). Moreover, the analysis will be repeated for the per protocol population, consisting of patients who sufficiently adhered to the study protocol. There are subgroup analyses planned based ALSFRS-R slope per month (< / ≥ median and < / ≥ upper third, both pre-baseline and during the study), NfL baseline values (< / ≥ median and < / ≥ upper third), NfL slopes (< / ≥ median and < / ≥ upper third during the study); patients with and without loss of BMI (both pre-baseline and during the study).

Safety: Adverse Events will be coded by MedDRA terminology and analyzed by evaluating absolute and relative frequencies. Vital signs and laboratory values will be analyzed by the two-sample t-test or Wilcoxon rank sum test and sample characteristics.

Secondary endpoints: Descriptive statistics will be provided. For continuous outcomes, the two-sample t-test or the Wilcoxon rank sum test will be used for the investigation of group differences as appropriate. For group differences of survival, Kaplan Meier curves and the log-rank test will be used. For ALSFRS-R, the slopes (loss of points per month) during treatment will be analyzed as well as the deviation of slopes from an NfL prediction model (Witzel et al.^33^).

Exploratory analysis of primary endpoint, all analyses of secondary endpoints and safety analysis are explorative at a significance level of 0.05 (two-sided).

All analyses will be performed by SAS 9.4.

**5.2 Power / Sample size considerations**

Since there are no systematic data about the clinical effect of ketone bodies in ALS, power calculation is based on the effect of a high-caloric fatty diet on NfL serum levels as shown in the LIPCAL-ALS study which, after logarithmic transformation of NfL values, found a mean NfL slope of -0.016 (SD 0.061) (pg/ml)/month for the fatty diet group and +0.019 (pg/ml)/month (SD 0.053) for the placebo group^17^. Assuming a treatment period of 6 months in the proposed study, the same effect size of 0.034, a one-sided significance level of 5%, a drop-out rate of 11% (5.5% deaths and 5.5% drop-outs for other reasons based on 6-month data from LIPCAL-ALS), and a power of 80%, a sample size of n=38 per treatment group will have to be analyzed. Furthermore, in order to guarantee sufficient power in case of unexpectedly high drop-out rates due to potential intolerability, we will recruit one more patient for every patient who drops out within the first 4 weeks of the study.

**6.	SIGNATURES**

The present study protocol was subject to critical review and has been approved in the present version by the persons undersigned. The information contained is consistent with:

- The current risk-benefit assessment of the study intervention.
- The moral, ethical, and scientific principles governing clinical research as set out in the Declaration of Helsinki and the principles of GCP so far applicable.

**Coordinating Investigator/Project Management**

Name

_______________	_____________________________________

Date	Signature

_______________

Date

**References**

1.	Brooks BR, Miller RG, Swash M, Munsat TL. El Escorial revisited: revised criteria for the diagnosis of amyotrophic lateral sclerosis. *Amyotrophic lateral sclerosis and other motor neuron disorders : official publication of the World Federation of Neurology, Research Group on Motor Neuron Diseases* 2000; **1**(5): 293-9.

2.	Ludolph A, Drory V, Hardiman O, et al. A revision of the El Escorial criteria - 2015. *Amyotrophic lateral sclerosis & frontotemporal degeneration* 2015: 1-2.

3.	Seljeseth YM, Vollset SE, Tysnes OB. Increasing mortality from amyotrophic lateral sclerosis in Norway? *Neurology* 2000; **55**(9): 1262-6.

4.	Miller RG, Mitchell JD, Moore DH. Riluzole for amyotrophic lateral sclerosis (ALS)/motor neuron disease (MND). *The Cochrane database of systematic reviews* 2012; **3**: CD001447.

5.	Desport JC, Preux PM, Truong TC, Vallat JM, Sautereau D, Couratier P. Nutritional status is a prognostic factor for survival in ALS patients. *Neurology* 1999; **53**(5): 1059-63.

6.	Marin B, Desport JC, Kajeu P, et al. Alteration of nutritional status at diagnosis is a prognostic factor for survival of amyotrophic lateral sclerosis patients. *Journal of neurology, neurosurgery, and psychiatry* 2011; **82**(6): 628-34.

7.	Desport JC, Preux PM, Magy L, et al. Factors correlated with hypermetabolism in patients with amyotrophic lateral sclerosis. *The American journal of clinical nutrition* 2001; **74**(3): 328-34.

8.	Holm T, Maier A, Wicks P, et al. Severe loss of appetite in amyotrophic lateral sclerosis patients: online self-assessment study. *Interactive journal of medical research* 2013; **2**(1): e8.

9.	Dupuis L, Oudart H, Rene F, Gonzalez de Aguilar JL, Loeffler JP. Evidence for defective energy homeostasis in amyotrophic lateral sclerosis: benefit of a high-energy diet in a transgenic mouse model. *Proceedings of the National Academy of Sciences of the United States of America* 2004; **101**(30): 11159-64.

10.	Dupuis L, Pradat PF, Ludolph AC, Loeffler JP. Energy metabolism in amyotrophic lateral sclerosis. *Lancet neurology* 2011; **10**(1): 75-82.

11.	Mariosa D, Beard JD, Umbach DM, et al. Body Mass Index and Amyotrophic Lateral Sclerosis: A Study of US Military Veterans. *American journal of epidemiology* 2017; **2**: 1-10.

12.	Vielhaber S, Kunz D, Winkler K, et al. Mitochondrial DNA abnormalities in skeletal muscle of patients with sporadic amyotrophic lateral sclerosis. *Brain : a journal of neurology* 2000; **123**(Pt 7): 1339-48.

13.	Paganoni S, Deng J, Jaffa M, Cudkowicz ME, Wills AM. Body mass index, not dyslipidemia, is an independent predictor of survival in amyotrophic lateral sclerosis. *Muscle & nerve* 2011; **44**(1): 20-4.

14.	Dorst J, Cypionka J, Ludolph AC. High-caloric food supplements in the treatment of amyotrophic lateral sclerosis: A prospective interventional study. *Amyotrophic lateral sclerosis & frontotemporal degeneration* 2013; **14**(7-8): 533-6.

15.	Wills AM, Hubbard J, Macklin EA, et al. Hypercaloric enteral nutrition in patients with amyotrophic lateral sclerosis: a randomised, double-blind, placebo-controlled phase 2 trial. *Lancet* 2014; **383**(9934): 2065-72.

16.	Dorst J, Dupuis L, Petri S, et al. Percutaneous endoscopic gastrostomy in amyotrophic lateral sclerosis: a prospective observational study. *Journal of neurology* 2015.

17.	Ludolph AC, Dorst J, Dreyhaupt J, et al. Effect of high-caloric nutrition on survival in amyotrophic lateral sclerosis. *Annals of neurology* 2019; **17**(10): 25661.

18.	Dorst J, Schuster J, Dreyhaupt J, et al. Effect of high-caloric nutrition on serum neurofilament light chain levels in amyotrophic lateral sclerosis. *Journal of neurology, neurosurgery, and psychiatry* 2020; **91**(9): 1007-9.

19.	Cedarbaum JM, Stambler N, Malta E, et al. The ALSFRS-R: a revised ALS functional rating scale that incorporates assessments of respiratory function. BDNF ALS Study Group (Phase III). *Journal of the neurological sciences* 1999; **169**(1-2): 13-21.

20.	Ludolph AC, Langen KJ, Regard M, et al. Frontal lobe function in amyotrophic lateral sclerosis: a neuropsychologic and positron emission tomography study. *Acta neurologica Scandinavica* 1992; **85**(2): 81-9.

21.	Steinacker P, Huss A, Mayer B, et al. Diagnostic and prognostic significance of neurofilament light chain NF-L, but not progranulin and S100B, in the course of amyotrophic lateral sclerosis: Data from the German MND-net. *Amyotrophic lateral sclerosis & frontotemporal degeneration* 2017; **18**(1-2): 112-9.

22.	Verde F, Silani V, Otto M. Neurochemical biomarkers in amyotrophic lateral sclerosis. *Current opinion in neurology* 2019; **32**(5): 747-57.

23.	Kim DY, Rho JM. The ketogenic diet and epilepsy. *Curr Opin Clin Nutr Metab Care* 2008; **11**(2): 113-20.

24.	Grammatikopoulou MG, Goulis DG, Gkiouras K, et al. To Keto or Not to Keto? A Systematic Review of Randomized Controlled Trials Assessing the Effects of Ketogenic Therapy on Alzheimer Disease. *Adv Nutr* 2020; **29**(10).

25.	Phillips MCL, Murtagh DKJ, Gilbertson LJ, Asztely FJS, Lynch CDP. Low-fat versus ketogenic diet in Parkinson's disease: A pilot randomized controlled trial. *Movement disorders : official journal of the Movement Disorder Society* 2018; **33**(8): 1306-14.

26.	Zhao Z, Lange DJ, Voustianiouk A, et al. A ketogenic diet as a potential novel therapeutic intervention in amyotrophic lateral sclerosis. *BMC Neurosci* 2006; **7**(29): 1471-2202.

27.	Hashim SA, VanItallie TB. Ketone body therapy: from the ketogenic diet to the oral administration of ketone ester. *J Lipid Res* 2014; **55**(9): 1818-26.

28.	Stubbs BJ, Cox PJ, Evans RD, et al. On the Metabolism of Exogenous Ketones in Humans. *Front Physiol* 2017; **8**(848).

29.	Clarke K, Tchabanenko K, Pawlosky R, et al. Kinetics, safety and tolerability of (R)-3-hydroxybutyl (R)-3-hydroxybutyrate in healthy adult subjects. *Regul Toxicol Pharmacol* 2012; **63**(3): 401-8.

30.	Poffé C, Ramaekers M, Van Thienen R, Hespel P. Ketone ester supplementation blunts overreaching symptoms during endurance training overload. *J Physiol* 2019; **597**(12): 3009-27.

31.	Wilson MM, Thomas DR, Rubenstein LZ, et al. Appetite assessment: simple appetite questionnaire predicts weight loss in community-dwelling adults and nursing home residents. *The American journal of clinical nutrition* 2005; **82**(5): 1074-81.

32.	Rohlmann F MR, Goldschmidt L. Randomisation program ROM. *DVMD Conference 2004M; see Schweizer, B et al (ed): Dokumentation - der Schritt ins 3 Jahrtausend 8 DVMD-Tagung in Ulm, 1 - 2 April 2004 Universitätsverlag Ulm 2004* 2004.

33.	Witzel S, Frauhammer F, Steinacker P, et al. Neurofilament light and heterogeneity of disease progression in amyotrophic lateral sclerosis: development and validation of a prediction model to improve interventional trials. *Transl Neurodegener* 2021; **10**(1): 021-00257.
